# German soils affect biomass production, elemental profiles, and anti-inflammatory activity of three medicinal plants used in Brazilian traditional medicine: *Scoparia dulcis* L., *Physalis angulata *L., and *Porophyllum ruderale* (Jacq.) Cass.

**DOI:** 10.1038/s41598-026-56323-w

**Published:** 2026-06-18

**Authors:** Enrique B. Hernández, Philipp Gabor, Franziska Schanbacher, Vanessa Gisele Pasqualotto Severino, Wilson Mozena Leandro, Sabry M. Shaheen, Matthias Melzig, Alexander Weng, Jörg Rinklebe

**Affiliations:** 1 School of Architecture and Civil Engineering, Institute of Foundation Engineering, Water- and Waste-Management, Laboratory of Soil- and Groundwater-Management, University of Wuppertal, Pauluskirchstraße 7, 42285 Wuppertal, Germany; 2 Institute of Pharmacy, Freie Universität Berlin, Königin-Luise-Str. 2+4, 14195 Berlin, Germany; 3 Federal University of Goiás, University Campus, Esperança Avenue, Goiânia, Brazil

**Keywords:** Medicinal plants, Trace elements, Rare-earth elements (REEs), THP-1, Macrophage differentiation, Mass spectrometry, Environmental sciences, Plant sciences

## Abstract

Cultivated medicinal plants are an important source of bioactive raw materials. However, information on their performance in non-native soils and their associated elemental and pharmacological profiles is limited. This study evaluated whether the three South American medicinal species – *Physalis angulata* L., *Porophyllum ruderale* (Jacq.) Cass., and *Scoparia dulcis* L. – can be cultivated on two German soils (clayey silt and sandy loam) under greenhouse conditions, focusing on biomass production, elemental profile, and anti-inflammatory potential. Soil and plant tissues were analyzed for macro-, micro-nutrients, and trace elements, including rare earth elements (REEs), using inductively coupled plasma mass spectrometry (ICP-MS) and inductively coupled plasma optical emission spectrometry (ICP-OES). Crude extracts were tested for anti-inflammatory activity in lipopolysaccharide-stimulated THP-1-derived macrophages. High-resolution mass spectrometry with class-level annotation was used to characterize major natural product classes. The three plant species produced satisfactory above-ground biomass, with *P. ruderale* yielding the highest biomass, and nutrient levels were within ranges considered adequate for plant growth. Potentially toxic elements (Ni, Cu, Zn, and REEs) were detected at subtoxic concentrations. REEs were largely retained in the roots, with limited translocation to the shoots, which supports the safe use of the above-ground biomass. Acetone extracts of *P. angulata*, enriched in limonoids (physalins), exhibited the strongest inhibition of pro-inflammatory cytokine secretion. In contrast, *S. dulcis* extracts were dominated by diterpenoids, exhibiting solvent-dependent activity. Overall, both soils supported the production of pharmacologically active biomass, demonstrating their potential as a reliable raw material for this purpose in a Central European agricultural setting.

**Supplementary Information:**

The online version contains supplementary material available at 10.1038/s41598-026-56323-w.

## Introduction

Medicinal and aromatic plants are a valuable source of raw materials for industrial applications such as pharmaceuticals and cosmetics, contributing to sustainable economic growth through managed cultivation. Adaptation of these plants to non-native soils is crucial to optimize biomass yield suitable for industrial processing. Many medicinal plants are native to tropical ecosystems and are suitable for agricultural production in Europe. However, their cultivation in non-native regions requires agronomic strategies and protection systems against climatic adversities (drought, drastic changes of temperature, etc.) to achieve reliable yields, standardized phytochemical quality, and compliance with safety expectations under Good Agricultural and Collection Practices (GACP)^1^. The quality of the plants and their potential use in products is also compromised by the presence of toxic elements in the substrate and other potentially adverse soil characteristics^2^.

Plant-derived pharmaceuticals and cosmetics are perceived as products with fewer adverse effects given that their raw materials originate from natural sources. However, the raw materials are sometimes obtained from plants grown in their natural wild environment, where they are exposed to several anthropogenic or natural contaminants that may affect their pharmacological properties and safety profile^3^. Environmental conditions and soil quality significantly influence the elemental composition and biomass quality of these plants, which is critical for their reliable use as industrial raw materials^4,5^. N, P, K, Ca, S, Mg, Zn and Se are essential elements for plant nutrition. A deficiency of any of those elements may lead to chlorosis, inhibited plant growth, low agricultural productivity and delayed maturity among other effects^6^. Essential micronutrients also play an important role in enzymatic processes and metabolism in plants. Cu and Fe are mostly necessary for the process of photosynthesis, the metabolism of N, and for oxidative phosphorylation^7^. Mn, Ni, and Zn function in plants mainly in the formation of specific enzymes and the activation of enzymatic processes. The balance of these elements in their bioavailability and mobility in soil solution needs to be managed for optimal biomass production^7^.

Rare earth elements (REEs) also have an important function in biological processes of plants. Ce and La increase the photosynthetic rate by enhancing the mechanisms responsible for light use in leaves^8^. In low concentrations (~ 40 mg dm^−3^), these two elements have also been shown to support mycorrhizal relationships in the rhizosphere of some species, as a result showing significant improvement in plant growth^9,10^. Concentrations of Y under 2.5 µM improve photosynthesis and reduce the negative effects caused by Ni toxicity in plants^11^. Nd has been shown to enhance biomass production and also improves the assimilation of nutrients essential for the optimal development of plants^12^. In trace concentrations REEs are beneficial for plants, while high concentrations of those elements have adverse effects on plant growth, seed germination, photosynthesis and plant metabolism^13,14^.

*Physalis angulata* L. is a neotropical species used in Brazilian traditional medicine for rheumatic and skin-related inflammatory disorders^15^, and it has been extensively studied due to its rich content in active secondary metabolites. Natural steroids such as physalins and withaphysalins have been isolated from above-ground plant parts and fruits. Many of these compounds have potent immunomodulatory effects, making this plant a valuable source of bioactive compounds^16–19^. Additionally, *P. angulata* shows strong adaptability to diverse conditions, and its seeds germinate over a wide range of temperatures, pH, osmotic and salinity conditions, indicating a high capacity to establish in arid and semi-arid environments^20,21^.

*Scoparia dulcis* L. contains a variety of bioactive compounds including several previously identified diterpenoids^22^. In Brazilian folk medicine, *S. dulcis* has been used for skin wounds, haemorrhoids, and other inflammatory conditions^23^. Most of the evidence for the plant’s anti-inflammatory potential was derived using crude extracts tested in in vivo models^24–26^, while one study reported inhibition of specific inflammatory pathways by isolated compounds in an in vitro model^27^. *S. dulcis* is originally from South America, but it is now common throughout tropical and subtropical regions of Asia and Africa, demonstrating its broad ecological adaptability^28^.

*Porophyllum ruderale* (Jacq.) Cass. contains diverse phenolic compounds that drive its anti-inflammatory and antinociceptive activity^25,29^. In Brazilian traditional medicine, P. ruderale is used for bruises and other inflammatory conditions^30^. However, the total phenolic content of the plant is strongly influenced by the characteristics of the soil and agroclimatic zones, underscoring the need for cultivation protocols that stabilize composition^31^. Despite this sensitivity, *P. ruderale* is native to Mexico and South America but has naturalized in tropical and subtropical regions worldwide, demonstrating ecological plasticity and suitability for cultivation outside its native environment^32^.

The THP-1 cell line, derived from human monocytic leukemia, is a widely used tool in immunological research as it can be differentiated into a phenotype that closely resembles the characteristics of primary macrophages. The simplicity and reproducibility of this model have contributed significantly to its widespread use in activity screening for potential anti-inflammatory drugs^33^. These bioassays evaluate the anti-inflammatory potential of crude extracts, which is important for determining the added value of harvested biomass for industrial use.

*P. ruderale*, *S. dulcis,* and *P. angulata* were selected for this study due to previous reports on their anti-inflammatory properties, when grown in native soils^17–19,22,25,31,34,35^ and the availability of their seeds on the European market. Previous studies have examined how soil properties influence the phytochemical composition of medicinal plants and the cultivation of tropical species outside their native habitats. However, these aspects have rarely been assessed together, especially with regard to Central European soils. Additionally, the existing literature on *P. angulata*, *P. ruderale*, and *S. dulcis* primarily uses in vivo models or cell-free assays for pharmacological evaluations, limiting their direct relevance to human immune responses. To the best of our knowledge, there are no data on cultivating these species in German soils or on the anti-inflammatory activity of their extracts in a human-relevant macrophage model. The present study addresses these gaps. It integrates elemental profiling **–** including rare earth element dynamics, which have received little attention in the context of medicinal plant cultivation **–** with the evaluation of pharmacological activity in lipopolysaccharide (LPS)-stimulated THP-1-derived macrophages. This allows for a direct assessment of whether soil properties in a non-native Central European environment compromise the pharmaceutical quality of harvested biomass.

The aims of the current study were to: (1) determine the effect of two different soils originating from Germany on the above-ground biomass production in *P. angulata*, *P. ruderale*, and *S. dulcis*; (2) elucidate the uptake and translocation of essential nutrients, trace elements and rare-earth elements in the plant body; (3) evaluate crude extracts for anti-inflammatory activity in a THP-1-derived human macrophage inflammatory response model; and (4) compare the composition of active extracts at the natural product class level.

## Materials and methods

### Soil sampling and soil treatment

Soil was collected from an agricultural area in Mülheim an der Ruhr (51°23′46.6"N, 6°52′47.6"E) and in Düsseldorf (51°15′12.0"N, 6°54′29.4"E), Germany. Soil sampling was performed from a depth of 0 to 15 cm at each location. All soil samples were then air-dried, sieved (< 2 mm), well homogenized, and stored until further analysis.

A 65% water saturation in soil was considered to be optimal for plant development. To estimate the 65% water saturation of soil, the water holding capacity of each soil was calculated by saturating 100 g of each soil (air-dried and sieved < 2 mm) with water applied through a pipette. Then, considering the volume of water required to saturate each soil 100%, the 65% saturation was calculated by simple cross-multiplication. The 65% water saturation of the soil collected from Düsseldorf was estimated to be 837.14 mL water/pot and for the soil from Mülheim an der Ruhr 418.57 mL water/pot.

## Implementation and treatments

The experiment was carried out in a greenhouse in Wuppertal, Germany (51°15′33"N, 7°09′57"E) from May 15^th^ until July 26^th^, 2023. The three plant species *P. angulata, P. ruderale*, *and S. dulcis*, used in traditional Brazilian medicine, were cultivated in two different soils from Germany (collected from Düsseldorf and from Mülheim an der Ruhr).

Plant seeds of *P. ruderale* and *S. dulcis* were obtained on the European market from Templiner Kräutergarten (Templin, Germany), where these species are propagated from established cultivation stock. Plant seeds of *P. angulata* were obtained from Sunshine-Seeds (Ahlen, Germany) who sources this species from suppliers in the United States of America. All seeds were germinated under controlled conditions in the laboratory. The seedlings were planted in plant pots (of 4 L volume capacity) containing 4058 g soil (dry weight) each. Thirteen seedlings per pot were transplanted and four replicates were used for each combination of plant species and arable soil. In total 24 pots were used, of which 12 contained each plant species in the soil collected in Düsseldorf and the other 12 pots contained each plant species in the soil collected in Mülheim an der Ruhr. The plants were watered twice a week until each pot reached 65% water saturation in soil. Water was administered to the pots by using a graduated glass beaker. No fertilizer was added to the pots. During the experimental phase, the temperature inside the Greenhouse was measured with a thermometer twice a week to track fluctuations. The temperature measurement ranged from 15 to 31 °C during the period of the experiment. The sunlight exposure of the plants was between 8 to 10 h per day. This is very similar to the native environmental conditions of the plants.

## Analysis of soil samples

The soil pH was measured in a suspension of Calcium chloride (CaCl_2_). at a ratio of 1:1. The electrical conductivity was determined according to ISO 11,265^36^. Total carbon content was measured using a CN analyzer (multi N/C 2100 S Analytic Jena). For this, 1 g of sieved soil (< 2 mm) was spiked with 500 mL of 10% HCL and dried at 60 °C for one hour. Once the soil was dry, total carbon content was measured^37^. Soil texture was determined using the PARIO Automated Soil Particle Size Analysis System. The PARIO system applies an integrated gravity sedimentation analysis based on a suspension pressure method for particle size determination as described by Durner et al.^38^. PARIO plotted results of the texture analysis automatically on the triangle of the German soil texture classification and also provided the proportions of coarse sand (630–2000 µm), middle sand (200- 630 µm), fine sand (63- 200 µm), coarse silt (20–63 µm), middle silt (6.3–20 µm), fine silt (2.0–6.3 µm) and clay (< 2.0 µm) in the soil samples.

To determine the total concentration of Ca, Ce, Cu, Fe, K, La, Mg, Mn, Nd, Ni, P, S, Y, and Zn in soil, the soil was extracted using a mixture of 65% HNO_3_ (Fisher Scientific, d = 1.40, N/2185/PB15, Belgium) and 30% H_2_O_2_ (Supelco, Merck KGaA, Germany) in well-sealed vessels in an ETHOS.start microwave system. Then, the solution was filtered through filter paper (150 mm, 8 μm particle retention) and the samples were stored until measurement.

One soil sample was collected from each pot with the use of a ceramic knife. The sampled soil was then air-dried and used to extract with ammonium bicarbonate-diethylenetriaminepentaacetic acid (AB-DTPA) the mobile fraction of Ca, Ce, Cu, Fe, K, La, Mg, Mn, Nd, Ni, P, S, Y, and Zn. This extraction method was originally recommended for alkaline soils; however, Malathi and Stalin^39^ suggested that it is also suitable for elemental analysis in acidic soils.

## Preparation and analysis of plant samples

The plants were harvested after 9 weeks. The roots were subjected to three washings after being pulled out of the soil to remove soil residues. The first washing was done with tap water, then the roots were washed with a 32% HCl (Supelco, Merck KGaA, Germany) solution and deionized water (Milli-Q water) in a ratio of 1:29. The last washing was done with deionized water and after that the roots were air-dried together with the remaining plant parts in a ventilated room (+ 22 °C ± 3 °C) for four weeks. Afterwards ten plants per pot were selected to measure the dry above-ground biomass. The dry biomass was calculated by weighing the air-dried stem and leaves in an analytical laboratory scale KERN EW 420-3NM. The plant samples were then divided into stems, leaves and roots, cut into small pieces and then ground using metal free (agate) material in a Planetary Ball Mill (PM 200, Retsch GmbH) for 2 h. Afterwards, 0.5 g of each plant sample was weighed and dry-ashed in ceramic vessels in a muffle furnace (Nabertherm L5/P 330, Nabertherm GmbH) for 5 h at 550 °C. After the ashing process, the samples were dissolved in 50 ml of a solution of 32% HCl and deionized water (1:4), filtered through filter paper (150 mm, 8 μm particle retention) and stored until analysis.

## Element analytics

The measurements of total Ca, Cu, Fe, K, Mg, Mn, Ni, P, S, and Zn in soil and in plant samples and in the AB-DTPA extract were carried out using an optical emission spectrometry with inductively coupled plasma (ICP-OES, HORIBA Ultima Expert, HORIBA France SAS). The measurements of La, Nd, Ce and Y were performed with an inductively coupled plasma mass spectrometer (8900 ICP-MS/MS QQQ, Triple Quad, Agilent). The data quality control was ensured by calibrating both instruments with standard solutions (Merck) and by using blank samples. Only measurements which had a relative standard deviation (RSD) ≤ 20% were included in the analysis. All materials used during the process of analysis of the soil samples were made of plastic, glass, or ceramic in order to avoid contamination of the samples by metals from laboratory equipment. These data-quality control measures were also applied to the analysis of plant samples.

## Statistical analysis

RStudio version 4.3.2 (Posit, 2023) was used for the statistical analysis of the data on soil elements, elements in plant tissue, and biomass. A Kruskal–Wallis test was used to evaluate the influence of the two soils on the above-ground biomass production, and the uptake and translocation of elements. This test was selected due to its sensitivity to outliers, which provides a better analysis of the dataset. For all significant Kruskal–Wallis tests, a post hoc Dunn’s multiple comparison test with Bonferroni correction was applied to identify significant effects. The significance level chosen for the analysis was 5% (α = 0.05). The data of Ca, K, Mg, P, S, Zn, Ni, Mn, Fe, and Cu inside plant tissue were winsorized at 80% to reduce the influence of outliers in the statistical analysis. This method was used for the treatment of outliers because it keeps the integrity of the data and accommodates valid extreme data points. Figures were created using the software OriginPro 2023.

## Extract preparation

Pooled above-ground plant parts cultivated in different soils were ground to a medium fine powder using a mill (IKA M20 universal mill). The powder was extracted with 70% ethanol (v/v) or acetone in a ratio of 1:5 for 24 h in a rotary shaker. The powder particles were then separated by centrifugation and the solvent was removed by vacuum centrifugation (SP Genevac miVAC). The preparations extracted with 70% ethanol (v/v) were additionally lyophilized (Christ Alpha 2–4) to remove residual water. The weight of the remaining extract was determined to calculate the extract yield. To obtain stock solutions for in vitro assays, the extracts were dissolved in dimethyl sulfoxide (DMSO) at 100 mg/mL, sterile-filtered, aliquoted and stored at −20 °C.

## Culture of THP-1 cell line

The human monocytic leukemia cell line THP-1 (ACC 16) was obtained from the German Collection of Microorganisms and Cell Cultures GmbH, Braunschweig, Germany. Cells were cultured in RPMI 1640 medium with GlutaMAX (Gibco, New York, USA) and supplemented with 10% fetal bovine serum (FBS) (Bio&Sell, Feucht, Germany) in T-75 flasks in a humidified incubator at 37 °C and 5% CO_2_. Subculturing was performed twice a week to maintain cell density within the recommended range of 1 × 10^5^ to 1 × 10^6^ cells/mL. Cultures were periodically tested for mycoplasma contamination using a mycoplasma PCR detection kit (Applied Biological Materials, Richmond, Canada).

## In vitro assay

To achieve differentiation of THP-1 cells, 2 × 10^5^ cells were seeded in each well of a 48-well multiwell plate in complete media supplemented with 50 ng/mL Phorbol 12-myristate 13-acetate (PMA) (Sigma Aldrich, Germany). After 48 h of incubation, the media was aspirated and the cells were gently washed three times with DPBS, followed by 24 h of incubation in complete media without PMA. Cells were treated with different concentrations of plant extracts, 0.1% (v/v) DMSO as vehicle control, 0.5 µM dexamethasone as positive control and pure medium as negative control. After 1 h of pre-treatment, 100 ng/mL lipopolysaccharide (LPS) from *E. coli* O111:B4 (Sigma Aldrich, Germany) was added from a stock solution. At least three technical replicates were performed for each condition. Initially, three technical replicates were performed at concentrations of 100 µg/mL and 10 µg/mL. Since no soil-dependent differences in bioactivity were observed, a second biological replicate was performed for extracts that significantly inhibited multiple cytokines, including the 1 µg/mL concentration. Samples for cytokine determination were collected from the culture supernatants after 4 h (TNF-α) and 24 h (IL-6, IL-1β), centrifuged and stored at − 70 °C until analysis. After removal of all supernatant, 200 µL of media containing 0.5 mM WST-8 (Abcr, Germany) and 40 µM menadione (Sigma Aldrich, Germany) was added to each well. The plate was then incubated for one hour before being scanned using a microplate reader at 450 nm with a reference wavelength of 600 nm. One well containing no cells was used as a blank and its value was then subtracted from all values. Values were normalized to the negative control of each assay plate. Statistical analysis was performed in GraphPad Prism 9 using the Kruskal–Wallis test and Dunn’s test as post hoc test.

## Determination of cytokine secretion

Cytokine concentrations in the supernatant were determined using commercially available ELISA kits for TNF-α, IL-1β and IL-6 (Cat. No.: 88–7261-77; 88–7066-77; 88–7346-77, Invitrogen, Life Technologies). All samples and standards were tested in duplicate according to the manufacturer’s instructions. Values were normalized to the LPS + control of each assay plate. Statistical analysis was performed in GraphPad Prism 9 using the Kruskal–Wallis test and Dunn’s test as post hoc test.

### Mass spectrometry acquisition and analysis

HRMS (high-resolution mass spectrometry) data were acquired using an Orbitrap Exploris 240 mass spectrometer equipped with a heated ESI interface coupled to a Vanquish Flex HPLC system (Thermo Fisher Scientific). The chromatographic conditions were as follows: Kinetex C18 column (50 × 2.1 mm, 2.6 µm, 100 Å, Phenomenex), gradient from 5 – 100% acetonitrile in H_2_O (each containing 0.1% formic acid) at 0.4 mL/min for 16 min, followed by 100% acetonitrile for 4 min. HRMS data acquisition: positive and negative ionization mode, ESI spray voltage 3.5 kV and − 2.5 kV, capillary temperature 350 °C, sheath gas flow rate 40 L/min, auxiliary gas flow rate 5 L/min. Full scan spectra were acquired from *m/z* 133.4 to 2000 with a resolution of 35.000 at *m/z* 200, automatic gain control (AGC) 5 × 10^5^, maximum injection time 120 ms. MS/MS spectra were acquired in data-dependent acquisition mode (dd-MS^2^), stepped collision energy of 30, 60, and 75 eV (resulting in 55 eV), a resolution of 17.500 at *m/z* 200, an AGC of 2 × 10^5^, and a maximum injection time of 75 ms. A TopN experiment (N = 5, loop count 5) was implemented for triggering the dd-MS2 acquisition. Raw MS data files were converted to .mzML files and filtered by polarity to separate positive and negative ion mode scans using msConvert (ProteoWizard, version 3.0). Only the positive mode data were used for analysis as it contained a larger amount of fragmentation, information leading to more detected features. Raw data processing was performed using MZmine (version 4.5) utilizing several modules for feature processing, alignment, grouping, and ion identity networking. The exact batch used for processing is provided as .mzbatch in supplementary files. Exported feature lists were annotated using SIRIUS (version 6.1.0) sub tools SIRIUS^40^, ZODIAC^41^, CSI:FingerID fingerprint prediction^42,43^, CANOPUS^44^, NPClassifier^45^ and CSI:FingerID structure database search. The exact computation parameters are supplied as supplementary files. Results were exported as .tsv files and merged with the feature quantification table created in MZmine. Results from features grouped by metaCorrelate and ion identity networking were merged in order to harmonize annotation results across different adducts and modifications. In cases where more than one adduct or in-source fragment was annotated, the feature with the highest confidence score was selected for assigning results to the remaining features from the group.

## Results

### Soil characteristics

Both soils differed in texture, total carbon, electrical conductivity, pH, and element content. The soil from Düsseldorf was dominated by silt (76.6% silt, 14.5% clay and 8.9% sand). The soil pH was 6.35, the electrical conductivity 53.5 µS/cm, and the total carbon content was 3.5%. In contrast, the soil from Mülheim an der Ruhr was dominated by sand (62.38% sand, 25.73% silt, and 11.89% clay). The soil pH was 5.94 and the total carbon content was 1.85%. The values of both parameters were lower than in the clayey silt soil from Düsseldorf. However, the soil electrical conductivity (95.3 µS/cm) was higher than in the clayey silt soil. Elements found in the clayey silt soil ranged from the highest total concentration to the lowest as follows: Fe > Ca > Mg > K > Mn > P > S > Zn > Ce > Ni > La > Nd > Cu > Y (Table 1). In contrast, the concentrations of the AB-DTPA extractable fractions of those elements followed a different order: Ca > Fe > P > Mg > K > Zn > S > Mn > Cu > Y > Nd > La > Ce > Ni. The total concentration of elements in the sandy loam soil from the highest to the lowest were Fe > Ca > K > Mg > P > Mn > S > Zn > Ce > Cu > Ni > La > Nd > Y. However, the AB-DTPA extractable concentrations of the elements followed the order Fe > Ca > P > K > Mg > S > Mn > Zn > Cu > Nd > Ce > Y > Ni > La. A statistical analysis performed of the AB-DTPA extractable fractions of the 14 elements in both soils showed that the concentrations of 12 elements differed significantly (p < 0.05). The content of AB-DTPA soluble Cu and Mn showed no significant difference between the two soils (p > 0.05; Table 1).

**Table 1:** Total content of elements and AB-DTPA-soluble elements in two German soils.

	Element content in clayey silt soil (Mean ± SD)		Element content in sandy loam soil (Mean ± SD)		
	Total content [mg kg^−1^ dm]	Extractable fraction in AB-DTPA [mg kg^−1^ dm]	Total content [mg kg^−1^ dm]	Extractable fraction in AB-DTPA [mg kg^−1^ dm]	*p*-values
**P**	710.29 ± 69.26	43.87 ± 12.06 a	649.16 ± 9.71	120.07 ± 10.78 b	0.04953
**Mg**	2821.47 ± 68.40	37.24 ± 5.79 a (Mg^2+^)	1984.35 ± 114.29	45.21 ± 0.96 b (Mg^2+^)	0.0495
**K**	2712.21 ± 81.10	37.16 ± 5.97 a (K^+^)	1988.05 ± 114.15	52.36 ± 1.75 b (K^+^)	0.04953
**Ca**	4121.41 ± 117.60	279.36 ± 84.64 a (Ca^2+^)	2279.45 ± 35.87	174.68 ± 2.15 b (Ca^2+^)	0.04953
**Ni**	28.44 ± 11.50	0.47 ± 0.03 a	14.57 ± 0.55	0.32 ± 0.02 b	0.0495
**Cu**	16.62 ± 0.58	4.9 ± 0.50 a	17.25 ± 0.42	4.99 ± 0.52 a	0.5127
**Fe**	19,390.61 ± 564.38	61.28 ± 1.67 a	16,173.81 ± 2275.41	370.99 ± 6.81 b	0.0495
**Mn**	1173.50 ± 4.22	8.43 ± 0.68 a (Mn^2+^)	539.94 ± 30.96	9.98 ± 1.37 a (Mn^2+^)	0.1266
**S**	269.64 ± 2.09	13.89 ± 1.24 a	227.73 ± 0.26	26.68 ± 4.57 b	0.0495
**Zn**	124.01 ± 2.81	25.16 ± 1.67 a	120.97 ± 10.44	9.15 ± 0.64 b	0.04953
**Y**	14.19 ± 0.79	2.31 ± 0.40 a	7.50 ± 0.48	0.43 ± 0.17 b	0.04953
**La**	24.76 ± 1.80	1.40 ± 0.23 a	12.65 ± 0.45	0.31 ± 0.12 b	0.04953
**Ce**	52.65 ± 3.52	1.30 ± 0.19 a	27.31 ± 1.18	0.43 ± 0.16 b	0.04953
**Nd**	23.91 ± 1.73	1.91 ± 0.34 a	11.82 ± 0.23	0.52 ± 0.19 b	0.04953

*dm = dry matter. Significantly different values (p < 0.05) are labeled with different letters.

### Above-ground biomass

**Fig. 1:**
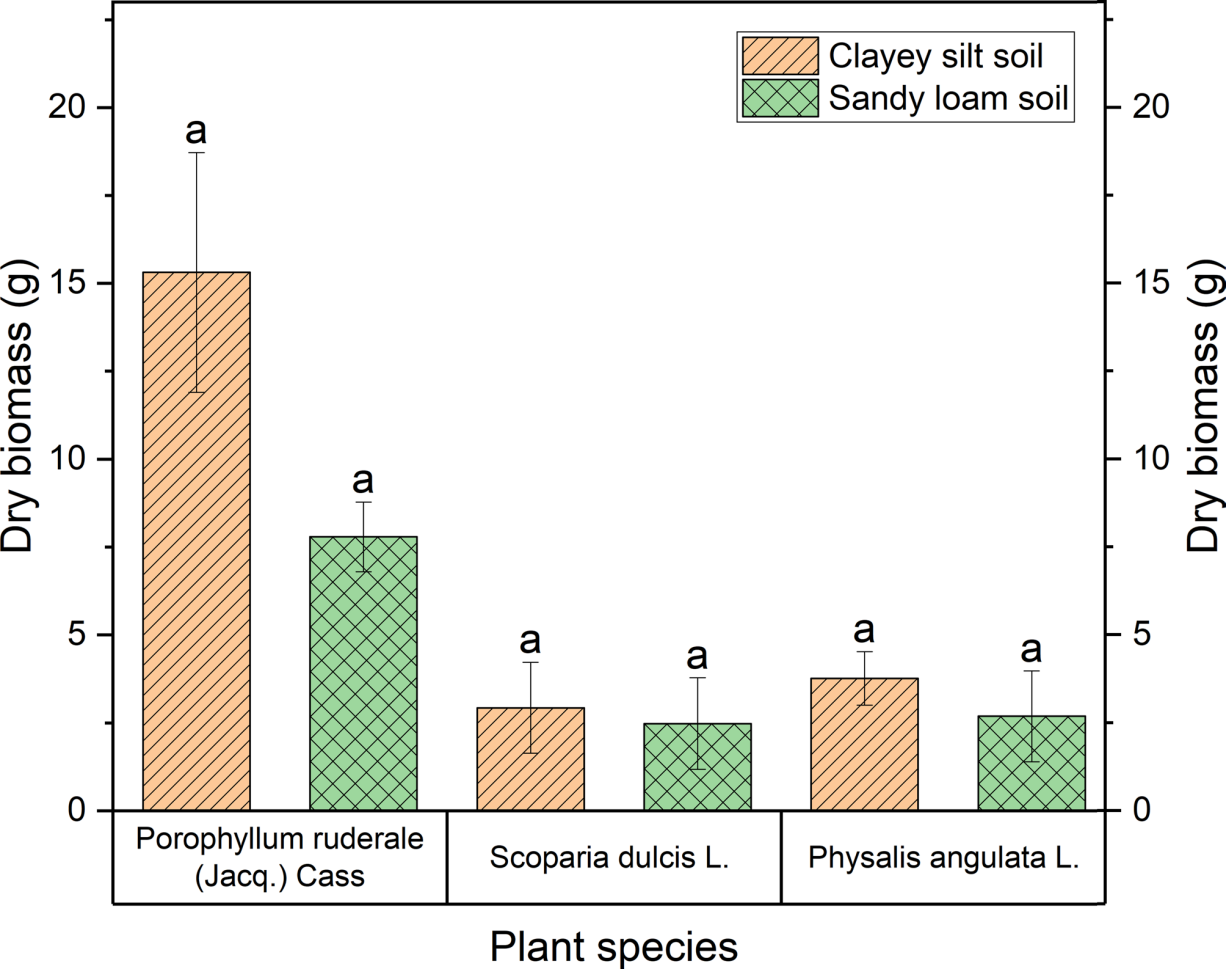
Above-ground biomass of *Porophyllum ruderale* (Jacq) Cass, *Scoparia dulcis* L. and *Physalis angulata* L. cultivated in two different German soils. The bars indicate the average ± SD above-ground biomass (stems and leaves) of the three cultivated plant species. Significantly different values (p < 0.05) are labeled with different letters.

*P. ruderale* produced the highest above-ground biomass yield, followed by *P. angulata*, and finally *S. dulcis*. Plants grown in the sandy loam soil produced slightly lower yields than those grown in the clayey silt soil, but this difference was not statistically significant (p ≥ 0.05; Fig. 1).

### Concentration of nutrients, trace elements and rare-earth elements in plant samples

**Fig. 2:**
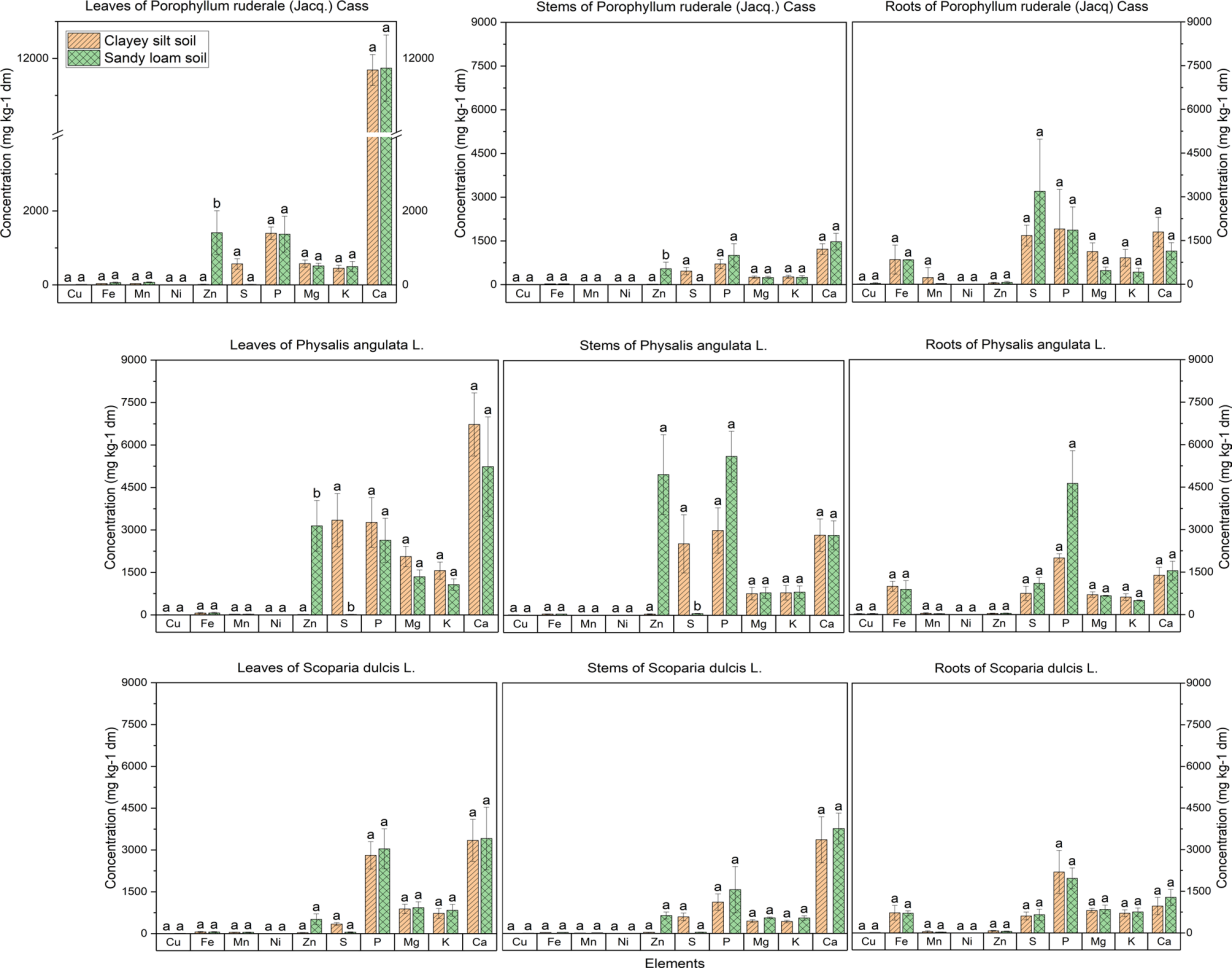
Concentration of nutrients and trace elements in plant parts of *Porophyllum ruderale* (Jacq) Cass, *Scoparia dulcis* L. and *Physalis angulata* L. cultivated in two different German soils. The bars indicates the average ± SD of elements in mg kg^−1^, dry matter. Significantly different values (p < 0.05) are labeled with different letters. The most relevant statistically significant effects of soils on plants were found in leaves of *P. ruderale* for Zn (*P* = 0.03496) and in leaves of *P. angulata* for Zn (*P* = 0.00499) and S (*P* = 0.03472).

While *P. angulata, P. ruderale,* and *S. dulcis* showed slightly reduced growth in sandy loam soil, this was not accompanied by accumulation of potentially toxic elements. The Zn concentration in the leaves of *P. ruderale* and *P. angulata* differed significantly between the plants grown in clayey silt soil and those grown in sandy loam soil, p < 0.05 (Fig. 2). Also, *S. dulcis* grown in the sandy loam soil showed a higher concentration of Zn (mg kg^−1^) in the leaves compared to its counterpart grown in the clayey silt soil, but the effect of the soils on the Zn concentration in *S. dulcis* is not statistically significant. The content of Ca in the leaves of all plants was also very high. However, the plants showed no macromorphological indications of Ca toxicity. The measurements of Fe in the samples of dried leaves from all three studied plants showed Fe contents much lower than threshold values considered toxic. *P. angulata*, *P. ruderale* , and *S. dulcis* did not show symptoms related to Fe toxicity. Besides, there was no significant difference (p ≥ 0.05) between Fe contents in the leaves and stems of the plants grown in both soils. Ni concentrations in plants were between 1.2 and 2.72 mg kg^−1^. Those values were far below the tolerance limit (10 mg kg^−1^) at which plants start showing morphological symptoms of Ni toxicity. Cu concentrations in *P. angulata*, *P. ruderale*, and *S. dulcis* were below the tolerance limit of 30 mg kg^−1^.

The Bioconcentration Factor (BCF) calculated in the plants grown in the clayey silt soil showed that P and S were accumulated mostly in roots of *P. angulata*, *P. ruderale*, and *S. dulcis* (Table S1). Plant roots had P and S concentrations proportionally higher than Cu, Fe, K, La, Mg, Mn, Nd, Ni, Y, and Zn. *P. ruderale, P. angulata* and *S. dulcis* all showed high translocation of Ca to the leaves. *P. angulata* also showed a high translocation of K, Mg, and S to the leaves.

**Fig. 3:**
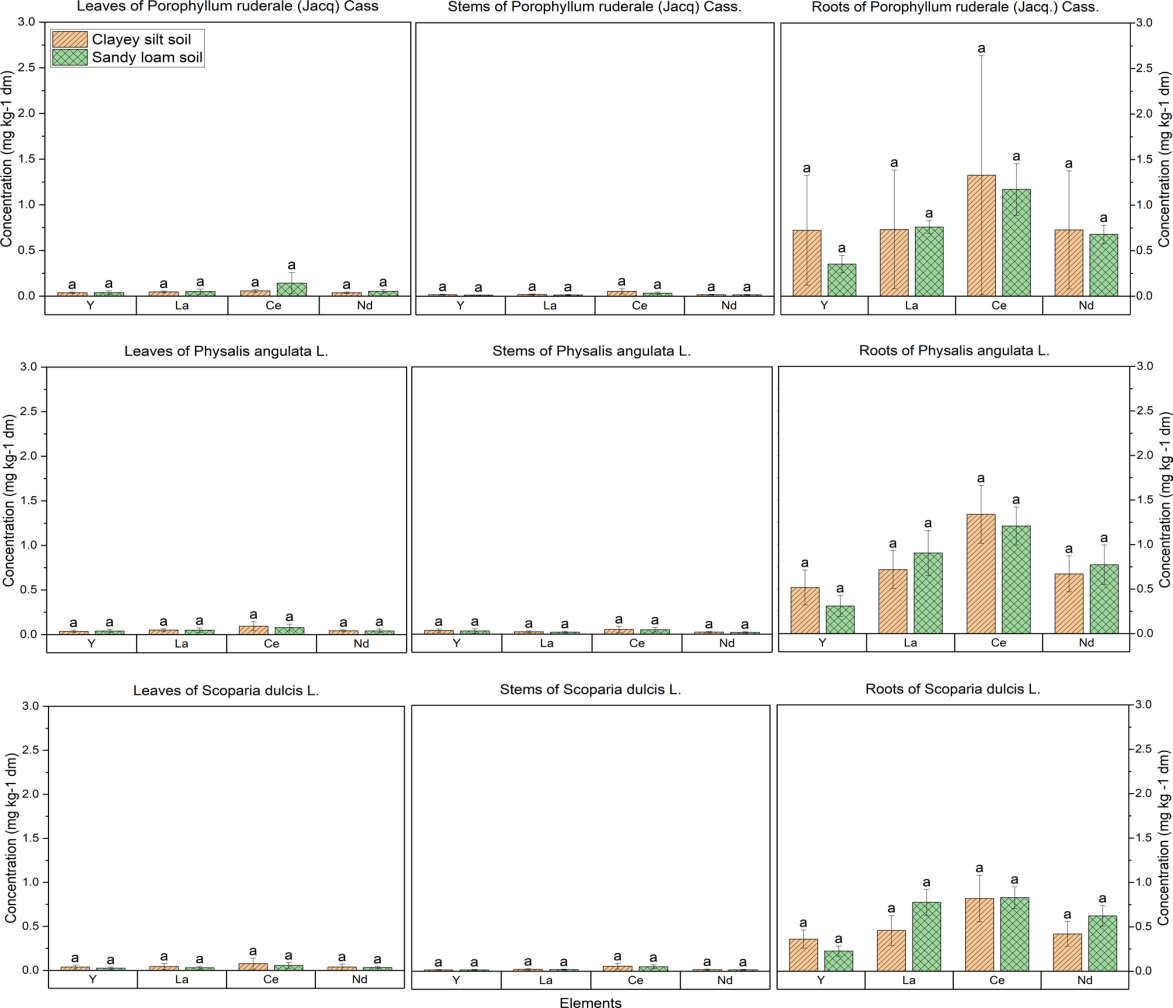
Concentration of rare-earth elements in plant parts of *Porophyllum ruderale* (Jacq) Cass, *Scoparia dulcis* L. and *Physalis angulata* L. cultivated in two different German soils. The bars indicate the average ± SD of elements in mg kg^−1^, dry matter. Significantly different values (p < 0.05) are labeled with different letters.

Overall, Ce was the rare-earth element with the highest concentration in each plant, followed by La (Fig. 3). All three plants showed no macromorphological symptoms of toxicity to REEs. *P. ruderale* , *P. angulata,* and *S. dulcis* mainly accumulated REEs in trace concentrations in the roots and showed low translocation to the above-ground plant parts.

### Anti-inflammatory activity in LPS-stimulated macrophages

**Fig. 4:**
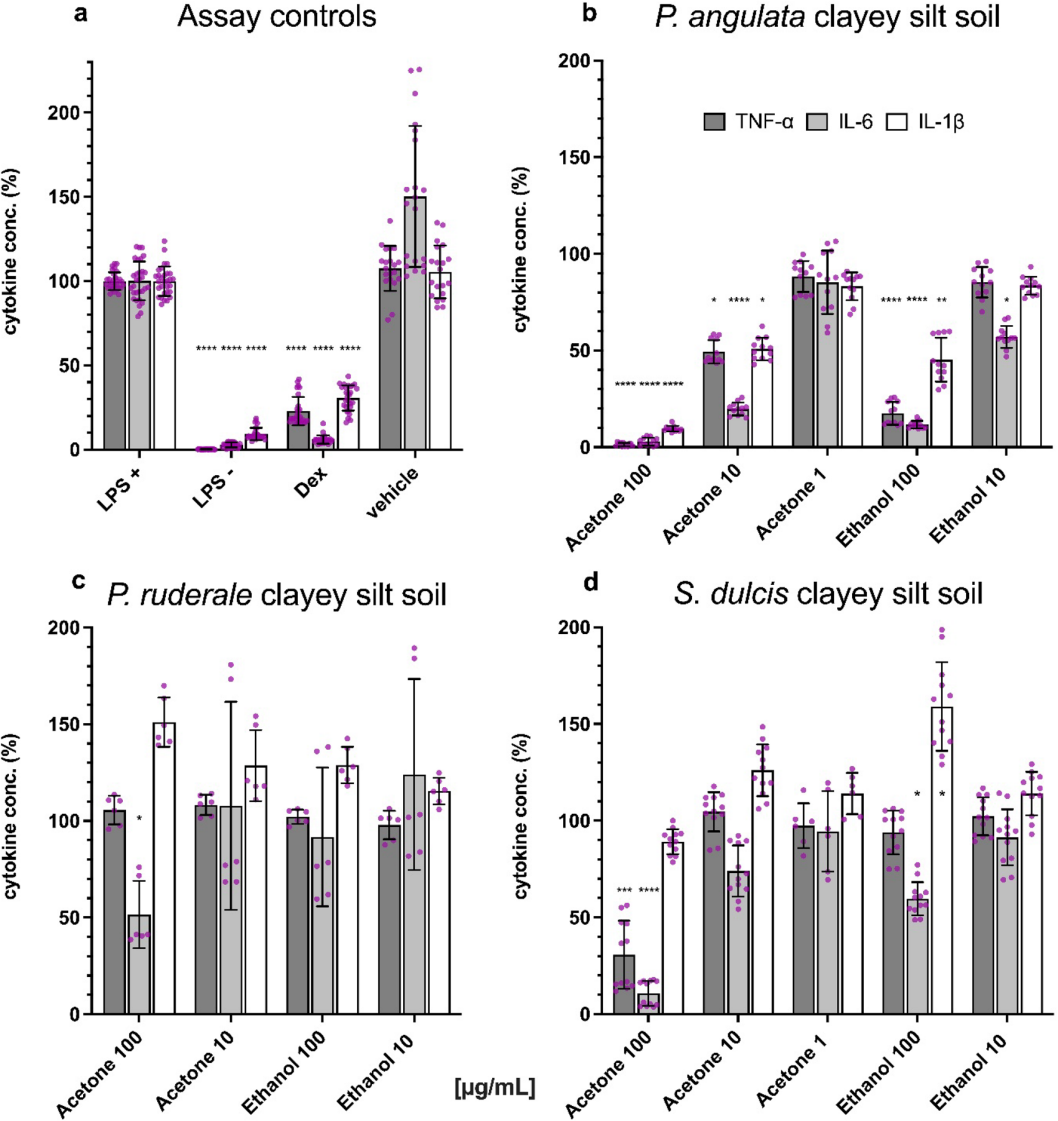
Inhibition of pro-inflammatory cytokine production by plant extracts in LPS-stimulated THP-1 macrophages. (**a**) pooled controls present on each assay plate. Dex: Dexamethasone 0,5 µM. vehicle: 0,1% DMSO. Data are normalized to the LPS + control and represent mean ± SD from three technical replicates quantified in duplicate via ELISA across 1 – 2 biological replicates. *p < 0.05, **p < 0.01, ***p < 0.001, ****p < 0.0001 compared to the LPS + control as determined using Kruskal–Wallis test with post hoc Dunn’s test.

To assess biomass quality for pharmaceutical applications, crude extracts were evaluated for anti-inflammatory activity using PMA-differentiated THP-1 macrophages stimulated with LPS as a functional bioassay. Cell viability was assessed using the WST-8 assay, which confirmed that the extracts did not impair cell health at the tested concentrations (Fig. S1). Upon stimulation with LPS, THP-1 derived macrophages showed an increase in secretion of pro-inflammatory cytokines such as TNF-α, IL-1β and IL-6. Dexamethasone, a common anti-inflammatory drug, was used as a positive control and significantly inhibited the secretion of all three cytokines (Fig. 4a).

Some crude extracts showed a concentration-dependent inhibition of pro-inflammatory cytokine secretion. The soil used for cultivation did not contribute to major differences in the activity of tested extracts (Fig. S2). In contrast, the two extractants used showed distinct differences in the degree of inhibition. Extracts from *P. angulata* made with acetone (PADA) and 70% ethanol (v/v) (PADE) both significantly inhibit the secretion of all three cytokines, but interestingly PADA showed greater reduction at all concentrations tested (Fig. 4b). In this in vitro model, PADA (at 100 µg/mL) had a stronger relative inhibitory effect on all three cytokines than dexamethasone (at 0.5 µM), though the observed effect should be interpreted cautiously due to differences in concentration units and the use of a crude plant extract versus a purified pharmaceutical compound. *S. dulcis* extracts prepared using acetone (SDDA) significantly inhibited TNF-α and IL-6 secretion, while SDDE only inhibited IL-6 secretion and caused a significant increase in IL-1β secretion (Fig. 4d). Of the *P. ruderale* extracts, only PRDA at the highest concentration showed significant inhibition of IL-6, whereas IL-1β secretion was increased slightly but not significantly (Fig. 4c).

### Analysis of extract composition using NPClassifier

To gain a deeper understanding of how the chemical composition of the extracts may lead to the observed differences in anti-inflammatory activity, HRMS data were acquired for extracts that significantly influence the secretion of multiple cytokines. Although computational methods for MS data processing have become increasingly accurate in correctly annotating known compounds from different classes, there are still some limitations. In particular, unreported compounds and isomeric compounds cannot be unambiguously annotated using standard techniques. We therefore analyzed the composition at the level of natural product classes using the SIRIUS sub-tool NPClassifier^45^, as class-level profiling is well suited for the rapid standardization of cultivation programs for medicinal plants.

The predominant classes in *P. angulata* extracts were limonoids and ergostane steroids. Within the limonoid class, multiple features could be tentatively assigned to physalins based on their m/z values and fragmentation patterns. This is consistent with the well-documented presence of this compound class in this species. However, it was not possible to unambiguously identify individual physalins for the majority of features due to the high structural similarity between isomers, which produces closely matched CSI:FingerID scores. For instance, the feature at m/z 527.191 [M + H]^+^ is present across three chromatographic peaks, with the top annotated candidates including Physalins A, F, G, N, X and Z, as well as Isophysalin A; however, no candidate achieved a sufficiently high discriminating score. Similar ambiguity was observed for the following features: m/z 511.197 [M + H]^+^ (Physalins B, C or Isophysalin B); m/z 545.202 [M + H]^+^ (Physalins D and E); m/z 511.197 [M-H₂O + H]^+^ (Physalins L, O, P and Y); and m/z 530.239 [M + NH₄]^+^ (Physalin M and Dihydrophysalin C). The only exception was the feature at m/z 563.167 [M + H]^+^, which could be confidently annotated as Physalin H, the only known chlorinated physalin, since no structural isomers of this compound have been reported. Features tentatively assigned to withaphysalins were found in the ergostane steroid class, including m/z 484.269 [M + NH₄]^+^ (Withaphysalin A or J), m/z 449.233 [M-H₂O + H]^+^ (Withaphysalin P) and m/z 500.265 [M + NH₄]^+^ (Withaphysalin M). However, unambiguous identification of individual physalins and withaphysalins would require authentic reference standards or additional orthogonal techniques, such as nuclear magnetic resonance (NMR). In acetone extracts, the content of limonoids was considerably higher, whereas extraction using 70% ethanol (v/v) yielded a higher content of flavonols and cinnamic acid derivatives (Table 2).

**Table 2:** Composition of *Physalis angulata* extracts by Superclass and class as annotated by NPClassifier.

**Superclass** / Class	Clayey silt acetone	Sandy loam acetone	Clayey silt ethanol	Sandy loam ethanol
**Triterpenoids**	**28,5%**	**29,7%**	**21,3%**	**21,3%**
Limonoids	25,3%	27,3%	19,3%	19,7%
**Steroids**	**12,4%**	**12,9%**	**11,9%**	**10,8%**
Ergostane steroids	9,6%	9,9%	8,9%	8,2%
Cholestane steroids	1,5%	1,7%	1,8%	1,7%
**Oligopeptides**	**5,6%**	**5,8%**	**6,8%**	**8,1%**
Cyclic peptides	1,1%	1,5%	1,8%	2,4%
Depsipeptides	1,6%	1,9%	2,3%	2,8%
**Glycerolipids**	**4,4%**	**3,7%**	**6,5%**	**4,5%**
Diacylglycerols	2,1%	1,8%	3,6%	2,3%
Glycosyldiacylglycerols	1,0%	0,9%	1,9%	1,4%
**Flavonoids**	**1,8%**	**1,9%**	**4,1%**	**5,3%**
Flavonols	1,2%	1,3%	3,3%	4,3%
**Phenylpropanoids (C6-C3)**	**0,8%**	**0,8%**	**2,9%**	**2,9%**
Cinnamic acids and derivatives	0,6%	0,6%	2,6%	2,6%
no annotation	11,4%	10,3%	11,4%	12,1%
sum superclasses	100,0%	100,0%	100,0%	100,0%

Values represent the summed peak areas of all features in each group shown as % of the total. Only Superclasses > 5% and classes > 1% were included.

**Fig. 5:**
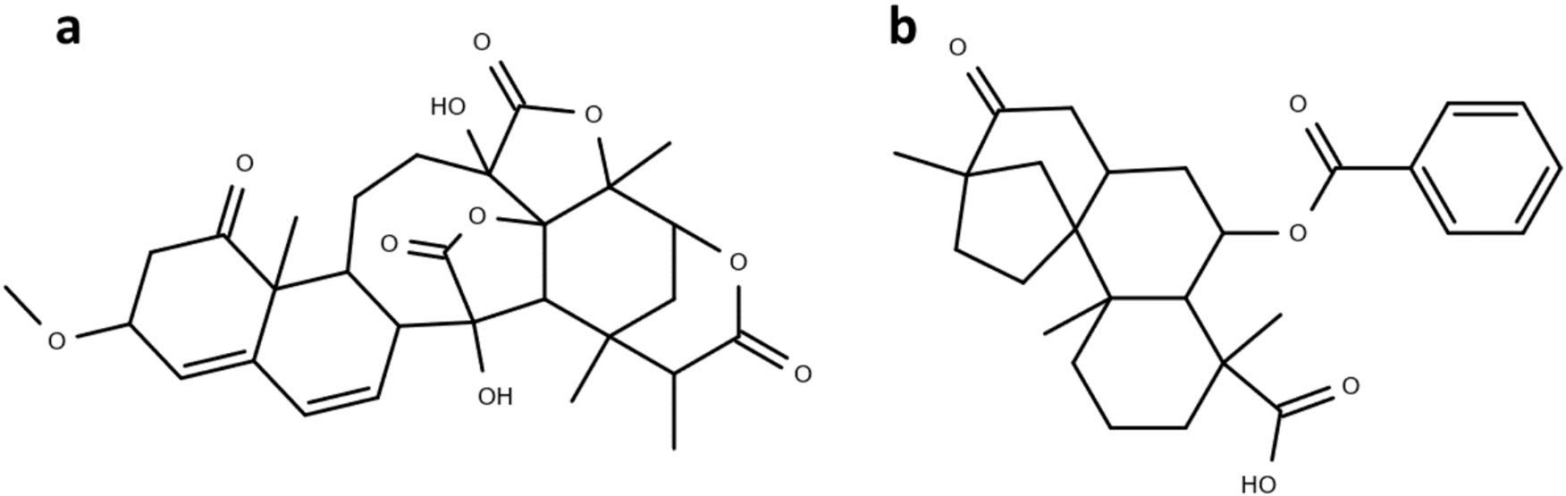
Representative chemical structures of the dominant natural product classes detected in the crude extracts of P. angulata and S. dulcis. (**a**) Physalin D (left) is a steroidal lactone and a representative of the limonoid class. This class was dominant in the acetone extracts of P. angulata. (**b**) Scopadulcic acid A (right) is a tetracyclic diterpenoid representative of the diterpenoid class, which dominated S. dulcis extracts. Due to structural similarities between isomers, individual compound identities within each class could not be unambiguously confirmed by HRMS annotation alone.

*S. dulcis* extracts were predominantly composed of diterpenoids belonging to various diterpenoid subclasses. Ethanolic extracts were composed of a smaller proportion of diterpenoids while flavones and glycerolipids were more abundant compared to extracts prepared using acetone (Table 3 and Fig. 5).

**Table 3:** Composition of *Scoparia dulcis* extracts by Superclass and class as annotated by NPClassifier.

**Superclass** / Class	clayey silt acetone	sandy loam acetone	clayey silt ethanol	sandy loam ethanol
**Diterpenoids**	**24,6%**	**24,0%**	**18,0%**	**18,5%**
Labdane diterpenoids	11,1%	9,2%	10,4%	10,7%
Kaurane and Phyllocladane diterpenoids	6,2%	6,5%	3,1%	3,2%
Beyerane diterpenoids	3,0%	2,6%	2,4%	2,5%
Tetracyclic diterpenoids	1,4%	1,2%	1,1%	1,1%
**Glycerolipids**	**13,5%**	**11,2%**	**18,9%**	**18,5%**
Triacylglycerols	7,8%	6,9%	10,0%	10,1%
Diacylglycerols	4,4%	3,4%	7,0%	6,7%
Glycosyldiacylglycerols	0,9%	0,6%	1,4%	1,3%
**Tryptophan alkaloids**	**10,0%**	**10,2%**	**8,1%**	**6,1%**
Pyrrole alkaloids	3,8%	4,7%	6,5%	4,9%
Pyrroloquinoline alkaloids	4,3%	4,1%	0,7%	0,6%
Carboline alkaloids	1,8%	1,2%	0,7%	0,5%
**Steroids**	**10,5%**	**9,5%**	**9,3%**	**11,4%**
Androstane steroids	5,2%	5,2%	5,1%	5,5%
Cholestane steroids	2,9%	1,8%	2,3%	4,3%
**Flavonoids**	**3,4%**	**3,8%**	**10,4%**	**11,1%**
Flavones	3,2%	3,7%	10,1%	11,0%
**Triterpenoids**	**5,9%**	**4,3%**	**3,9%**	**3,2%**
Lupane triterpenoids	1,4%	0,8%	0,9%	0,7%
Fusidane triterpenoids	1,3%	0,8%	1,0%	0,7%
**Sesquiterpenoids**	**4,4%**	**5,1%**	**2,2%**	**2,1%**
Longipinane sesquiterpenoids	1,3%	1,9%	0,3%	0,4%
Daucane sesquiterpenoids	0,8%	1,3%	0,1%	0,2%
no annotation	5,0%	5,2%	5,5%	5,2%
sum superclass	100,0%	100,0%	100,0%	100,0%

Values represent the summed peak areas of all features in each group shown as % of the total. Only Superclasses > 5% and classes > 1% were included.

## Discussion

### Soil characteristics

The element content in the clayey silt soil is higher due to the large surface area of silt and clay particles, which allows this soil to hold a higher amount of ions than the sandy loam soil^46^. The high proportion of silt and clay also allows the stabilization of organic carbon through the formation of nets of micro pores and aggregates that protect organic matter from decomposition^47^. These differences in texture, carbon content, and extractable nutrients are critical because they affect nutrient availability, biomass yield stability, and the safety of the harvested material. The broad standard deviation of Fe in the measurements might be caused by its high spatial variability (micro-variation), susceptibility to slight pH changes, and solubility^48^. Even when the soil was homogenized before the extraction, the samples and subsamples selected for analysis may vary widely in their Fe content.

### Above-ground biomass

The fertility of both soils might have influenced the biomass production of the plants. The high proportion of clay and silt particles increases the retention of organic carbon in the soil matrix due to a high specific surface area^49^. In addition to that, the stabilization of total carbon through clay minerals contributes to an increase in soil fertility due to a lower release of nutrients in comparison with sandy soils^47,50^. The low content of fine particles (silt and clay) in the sandy loam soil leads to a higher mobilization and leaching of elements than the clayey silt soil^51^. Also, the combined effect of an acidic pH and a sandy soil texture might enhance the breakdown rate of the labile carbon and leaching of elements to lower soil layers^52,53^.

Negative effects of P, Mg and Mn on biomass production are unlikely because their contents in both soils were within the optimal range suggested for plant development. The total concentrations of P, Mg and Mn in soils for optimal plant development are estimated to be: P = 200–3000 mg kg^−1^^54^, Mg = 500–5000 mg kg^−1^^55^, and Mn = 139–7780 mg kg^−1^^56^. Moreover, the values of the AB-DTPA extractable fraction of P, Mg and Mn in both soils suggest that the bioavailability of these elements was not a limitation for plant growth.

Critical levels of elements in soils vary depending on soil characteristics, species of vegetation and seasonal conditions^57^. By taking into consideration the soil characteristics and plant species, critical deficiency levels of total K and S in soil are estimated to range from 150 to 200 mg kg^−1^^58^ and from 13.5 to 14 mg kg^−1^^59^, respectively. The clayey silt soil and the sandy loam soil had a total K and S content above the critical level of deficiency. Also, the mobile fraction of K and S in soil and their content inside plant tissue suggested that deficiency of both elements was unlikely (Table 1 and Fig. 1). Overall, the levels of macronutrients and micronutrients in both soils were sufficient to support biomass production. This indicates that neither soil type imposes major nutritional constraints on these species.

### Concentration of nutrients, trace elements and rare-earth elements in plant samples

Despite their differences in the mobile fraction of elements in both soils, nutrient contents were within the range for optimal development for plants^54,55,60,61^.

The difference in the Zn concentration might be due to the slightly more acidic pH of the sandy loam soil and a low specific surface area of sand, which could have enhanced Zn mobilization and bioavailability. The difference in Zn accumulation in leaves could have influenced the biomass production, since Zn concentrations in leaves ranging from 100 to 700 mg kg^−1^ affect plant growth^62^. Zn surplus can alter the activity of enzymes involved in growth and photosynthesis, impairing nitrogen metabolism and potentially reducing biomass^63^.

Generally, the mobility of Ca in plant parts is low^64,65^. Nevertheless, the Ca absorbed through the root system is strongly translocated to the above-ground biomass of plants and accumulates in leaves^66,67^.

The content of Fe in the plant leaves suggests that an inhibitory effect of this element on the plant growth and biomass production is improbable. The threshold value of Fe at which plants show macromorphological symptoms of toxicity is considered to be around 500 mg kg^−1^ in dried leaves^68^. It is also worth noting that the tolerance limits of Fe in plants vary in different species^69^. Among the most common symptoms of Fe poisoning are leaf bronzing, chlorosis, darkening of roots, and reduction in plant growth^70,71^.

Inhibitory effects of elements such as Ni and Cu in the above-ground biomass production of *P. angulata, P. ruderale*, and *S. dulcis* are improbable. Ni deficiency was unlikely since it is required by plants in amounts lower than 0.5 mg kg^−1^^72,73^. The labile concentrations of Ni in the clayey silt soil and in the sandy loam soil were sufficient to supply the plants’ needs for this element. Symptoms of Cu toxicity become noticeable above concentrations of 30 mg kg^−1^, such as limited plant growth, chlorosis, reduced photosynthesis rate, and macromorphological alterations in the root system that reduce the absorption and transport of nutrients. This leads to low uptake, translocation and assimilation of essential elements such as P, Ca, Mg, Mn, and S in plants^74,75^.

The BCF suggested that most of the Ca absorbed by the plants was translocated and accumulated in the leaves. Ca follows that trend in many plant species^66^. The concentrations of K and Mg were seemingly balanced inside plant tissues. In case of imbalance among K and Mg in plant nutrition, K might induce Mg deficiency due to the antagonistic interactions of both elements^76^. The accumulation pattern of nutrients and trace elements in plant parts depends on the mobility of each element inside plant tissue and the physiology of each plant species. Many elements such as Pb are mainly accumulated in roots, while others such as Zn are highly translocated to leaves^77^.

In many plant species S is highly translocated to leaves to be assimilated into compounds such as cysteine, which is used for synthesis of several metabolites. Other S-containing compounds derived from plants, such as glucosinolates and organosulfur compounds have proven to have anti-inflammatory and anti-carcinogenic effects^78–81^. In the plants grown in the sandy loam soil S was mostly accumulated in roots in proportionally higher concentrations than all the other analyzed elements. High concentrations of K were found to be accumulated in roots of *P. angulata* and *S. dulcis.* However, Zn was the element with the highest concentration in each plant part of the three plant species in comparison to other elements. The content of Zn in the leaves of all plants surpassed the threshold value for Zn toxicity in leaves (100—700 mg kg^−1^)^62^. These Zn levels could contribute to the inhibited growth of these plants compared with their counterparts grown in the clayey silt soil (Table S2), suggesting that soil pH management or phosphorus fertilization could optimize biomass quality in this soil type^82^

The absence of macromorphological toxicity symptoms suggests that REEs accumulated in plant tissues at beneficial concentrations. Concentrations of Y higher than 25 mg kg^−1^in plant tissue cause oxidative stress, although it has also been shown to increase the biomass production in plants such as Triticum aestivum L^83^.. In the case of Ce and Nd, it is suggested that in many plant species their tolerance concentrations are near 50 mg kg^−1^ (Ce) and 100 mg kg^−1^ (Nd), respectively. Nd causes growth inhibition in plants when its total concentration in plant biomass exceeds 100 mg kg^−1^. Similar to Y, Ce can cause oxidative stress when its concentrations exceed species-specific tolerance limits. Other Ce related toxic effects reported in plants are reduction of plant yield and imbalance of ions such as Ca^2+^, K^+^, and Na^+^ due to alterations to the redox process in plants^84,85^. La also causes inhibition of biomass production, especially in shoots. The range in which plants have phytotoxic symptoms to La is 2.77—50 mg kg^−1^. However, the tolerance limit to this element varies in plant species^9^.

The accumulation of REEs in specific plant parts depends on the plant species. In the studied plants REEs mainly accumulated in trace concentrations in the roots and showed low translocation to the above-ground plant parts. This is consistent with previous results, which suggest that REEs are accumulated in trace amounts in plant tissues and that some species transport them to the stems and leaves only to a small extent to be integrated in different processes of plant metabolism^86^. Among the most important benefits of REEs for plants are: the stimulation of chlorophyll production, increase of fiber and protein content in fruits, regulation of the absorption of other minerals such as Cu and Fe, and inhibition of the absorption of heavy metals^14^. In high concentrations, REEs are toxic to plants. Therefore, the absorption of REEs through roots and their translocation to the above-ground plant parts is regulated by mechanisms such as ion-selective membranes in the root endoderm or limited availability of organic ligands in xylem to transport REEs upwards^77^. High concentrations of REEs in roots compared to stems and leaves are usually a sign of a very efficient filtering capacity by the endoderm. It retains most of the REEs on the cell wall and avoids their entrance to the cytoplasm. The particles that made their way into the cytoplasm can also face immobilization inside the xylem due to phosphate particles^14,87^. The mobilization of REEs in the cell walls of roots and leaves is also possible through regulated interactions and binding with Hydroxyl (-OH) and Carboxyl (-COOH) groups present in pectin. This is part of a detoxification mechanism of some plants, in which they make adjustments to the cell walls to fix REEs in pectine^88^. Plants such as Dicranopteris dichotoma (Thunb.) Bernh re-translocate REEs (bound to Low-Molecular-Weight Organic Acids) from stems and leaves back to the roots through the phloem as part of their detoxification system^89^. The regulated absorption of potentially toxic elements through the rhizosphere and their low translocation toward the above-ground parts reduce the risk of REE-related contamination, and support the safe and reliable use of plant material cultivated in Central European soils. In addition to that, some methods for the extraction of essential oils from aromatic/ medicinal plant reduce the concentration of trace elements in the final oil product^90,91^. This aligns well with the goal of the European Food Safety Authority (EFSA) of providing reliable information about the health risks of REEs in farming for a safer consumption of agricultural products^92^.

### Differential bioactivity and composition of extracts

The consistent activity across both soil types suggests that these plants can be reliably cultivated in Germany for pharmaceutical use, thereby reducing dependence on imports from tropical regions. The solvent-dependent differences have practical implications for processing of bioactive biomass. Acetone extraction yields more potent anti‑inflammatory extracts, making it preferable when the primary goal is to maximize the concentration of bioactive compounds, whereas 70% ethanol (v/v) offers an alternative with acceptable activity and process advantages for large‑scale use.

Aqueous extracts of *P. ruderale* have previously demonstrated anti-inflammatory properties in vivo by inhibiting leukocyte migration induced by intraperitoneal injection of carrageenan^25^. In vitro models using several polyphenolic compounds isolated from an aqueous extract have demonstrated inhibition of IL-8 and TNF-α secretion. The crude extract itself also showed activity when used at high concentrations^29^. Most likely, the previously reported anti-inflammatory activity could not be reproduced in this study due to the use of less polar extractants. As specifically the acetone extract (PRDA) inhibited IL-6 secretion, this suggests the presence of non-polar compounds in *P. ruderale* that might be responsible for its anti-inflammatory activity and warrant further targeted isolation.

The presence of various physalins in *P. angulata* has been well documented, and multiple physalins have previously demonstrated strong anti-inflammatory activity^16–19^. It therefore seems likely that the observed differences in the degree of cytokine inhibition can be attributed to the relative content of physalins present in the crude extracts. Although the precise identification of individual physalin isomers requires further analytical work, as detailed above, class-level characterization is sufficient for initial quality control in cultivation programs. These results indicate that *P. angulata* cultivated in German soils can serve as a relevant raw material for isolating natural substances with potent anti-inflammatory activity. However, the class-level resolution of the annotation approach does not allow for the absolute quantification of individual marker compounds. This limitation prevented the direct correlation of metabolite profiles with observed bioactivity. To establish such relationships, targeted quantification of specific physalins using authentic reference standards combined with a broader set of extract samples would be required, representing an important direction for future investigation.

Previously it was reported that several diterpenoids and flavones isolated from *S. dulcis* are capable of blocking the activation of NF-κB with IC_50_ values ranging from 10 to 100 µM. Diterpenoids had consistently lower IC_50_ values than tested flavones with one exception being 5-methylnobiletin^27^. It is likely that the lower total diterpenoid content is responsible for the varying inhibition of TNF-α and IL-6. What remains unclear is the significant increase in IL-1β secretion by SDDE, whereas SDDA does not affect the secretion of this specific cytokine. IL-1β secretion depends on caspase-1-mediated cleavage of pro-IL-1β downstream of NLRP3 inflammasome activation^93,94^. It is plausible that compounds from S. dulcis both inhibit NF-κB-mediated cytokine induction and, under certain extraction conditions, promote NLRP3 activation, leading to increased IL-1β secretion despite unchanged TNF-α and IL-6. S. *dulcis* contains several diterpenoids that have shown potent cytotoxicity against several cell lines^27,78,95^. Even if macrophage viability remains unchanged, as observed by the WST-8 assay, cytotoxic compounds can increase IL-1β secretion via activation of the NLRP3 inflammasome. Similarly, previous studies have shown that common chemotherapeutic drugs can induce IL-1β secretion in bone marrow-derived macrophages through activation of the NLRP3 inflammasome^96^. These results suggest that *S. dulcis* cultivated in non-native soils can be a promising source for the isolation of natural anti-inflammatory compounds. However, extraction conditions need to be standardized in order to reliably achieve effective extraction of bioactive compounds.

It is also important to note that the seeds used in this study were obtained from commercial suppliers outside of the native South American range of these species. There, the seeds were propagated under non-native conditions. *P. angulata* and *S. dulcis* showed clear anti-inflammatory activity in both soils and retained their characteristic bioactive compound classes*.* However, *P. ruderale* showed only marginal inhibition of IL-6 in crude extracts, which is consistent with previous reports indicating that its anti-inflammatory potential is primarily associated with isolated phenolic constituents rather than crude extract activity^29^. This suggests that their biosynthetic capacity is maintained under long-term cultivation outside their native range. This observation is directly relevant to the reliability of these species as sources of raw materials in Central European agricultural settings. However, comparison with autochthonous South American specimens under native soil conditions remains an important direction for future investigation.

The present study was designed as a controlled proof-of-concept investigation, and some limitations inherent to this design should be considered when interpreting the findings. While greenhouse conditions provided the necessary environmental control to isolate the effect of soil type on plant performance, they do not replicate the full range of variables present in open-field cultivation. These variables include seasonal temperature fluctuations, natural rainfall patterns, and biotic stress. Similarly, since the study only examined two soils from a specific region of western Germany over one growing season, the findings should be extrapolated to other soil types or climatic conditions with caution. Furthermore, meaningful correlation analyses between metabolite profiles and bioactivity would require targeted quantification of individual marker compounds using authentic reference standards and a larger set of extracts. Nevertheless, this controlled design represents a necessary and important first step toward establishing cultivation protocols for these species in Central European environments. Future multi-season field trials across a broader range of soil types would expand upon these findings and support the development of evidence-based cultivation guidelines.

## Conclusion

Both German soil types supported the cultivation of *P. angulata*, *P. ruderale*, and *S. dulcis* with satisfactory above-ground biomass production, and the nutrient profiles were within ranges considered adequate for plant growth. Potentially toxic elements, including rare earth elements, were largely retained in the roots, indicating that the above-ground biomass is safe for further use. Across different soil types, the anti-inflammatory activity remained consistent, being most evident in acetone extracts of P. angulata. The limonoid-dominated composition of these extracts aligns with the known bioactivity of physalins. These results suggest that Central European soils can support the production of pharmacologically relevant biomass from these South American medicinal species. This represents a step toward establishing evidence-based cultivation protocols in accordance with Good Agricultural and Collection Practices (GACP). However, future work involving multi-season field trials, targeted quantification of marker compounds, and comparison with autochthonous South American material is necessary to validate these findings on an agronomic scale.

## Supporting Information

Supporting figures, tables and method of calculation of BCF and TF in plants (DOC).

## Supplementary Information

Supplementary Information.

## Data Availability

The datasets generated during the current study are available from the corresponding author on reasonable request.

## Abbreviations

AB-DTPA: Ammonium bicarbonate-diethylenetriaminepentaacetic acid
BCF: Bioconcentration Factor
LPS: Lipopolysaccharide
PMA: Phorbol 12-myristate 13-acetate
IL-1β: Interleukin 1 beta
IL-6: Interleukin 6
TNF-α: Tumor necrosis factor alpha
NF-κB: Nuclear factor kappa-light-chain-enhancer of activated B cells
HRMS: High-resolution mass spectrometry
DMSO: Dimethyl sulfoxide
STAT-1/6: Signal transducer and activator of transcription 1/6
REE: Rare earth elements
PADA: *Physalis angulata* acetone extract clayey silt soil
PADE: *Physalis angulata* ethanol extract clayey silt soil
SDDA: *Scoparia dulcis* acetone extract clayey silt soil
SDDE: *Scoparia dulcis* ethanol extract clayey silt soil
PRDA: *Porophyllum ruderale* acetone extract clayey silt soil

## References

[1] Macaluso, D., Licciardo, F. & Carbone, K. Farming of Medicinal and Aromatic Plants in Italy: Structural Features and Economic Results. *Agriculture* **14**(1), 151. 10.3390/agriculture14010151 (2024).

[2] Fischer, A., Brodziak-Dopierała, B., Loska, K. & Stojko, J. The Assessment of Toxic Metals in Plants Used in Cosmetics and Cosmetology. *Int. J. of Environ. Res. and Public Health*10.3390/ijerph14101280 (2017). 10.3390/ijerph14101280 PMC5664780 29064437

[3] van Wyk, A. S. & Prinsloo, G. Health, safety and quality concerns of plant-based traditional medicines and herbal remedies. *S. Afr. J. Bot.* **133**, 54–62. 10.1016/j.sajb.2020.06.031 (2020).

[4] Dong, J. et al. Soil differentiation and soil comprehensive evaluation of in wild and cultivated Fritillaria pallidiflora Schrenk. *The Sci. of the Total Environ.* **872**, 162049. 10.1016/j.scitotenv.2023.162049 (2023). 10.1016/j.scitotenv.2023.162049 36804984

[5] Quan, M. & Liang, J. The influences of four types of soil on the growth, physiological and biochemical characteristics of Lycoris aurea (L’ Her.) Herb. *Sci. Rep.* **7**(1), 43284. 10.1038/srep43284 (2017). 28240308 10.1038/srep43284 PMC5327428

[6] Uchida, R. *Essential nutrients for plant growth: nutrient functions and deficiency symptoms* 31–55 (University of Hawaii at Manoa, 2000).

[7] Hänsch, R. & Mendel, R. R. Physiological functions of mineral micronutrients (Cu, Zn, Mn, Fe, Ni, Mo, B, Cl). *Curr. Opin. Plant Biol.* **12**(3), 259–266. 10.1016/j.pbi.2009.05.006 (2009). 19524482 10.1016/j.pbi.2009.05.006

[8] Lo Piccolo, E., Ceccanti, C., Guide, L. & Landi, M. Role of beneficial elements in plants implications for the photosynthetic process. *Photosynt.* **59**(2), 349–360 (2021).

[9] von Tucher, S. & Schmidhalter, U. Lanthanum uptake from soil and nutrient solution and its effects on plant growth. *Z. Pflanzenernähr. Bodenk.* **168**(4), 574–580. 10.1002/jpln.200520506 (2005).

[10] Vilela, L., Ramos, S., Carneiro, M. A., Faquin, V., Guilherme, L., Siqueira, O. Cerium (Ce) and Lanthanum (La) promoted plant growth and mycorrhizal colonization of maize in tropical soil. *Australian J Crop Sci*, **12**, 704–710. 10.21475/ajcs.18.12.05.PNE754, (2018).

[11] Lyu, K., Wang, X., Wang, L. & Wang, G. Rare-earth element yttrium enhances the tolerance of curly-leaf pondweed (Potamogeton crispus) to acute nickel toxicity. *Environ. Pollut.* **248**, 114–120. 10.1016/j.envpol.2019.01.120 (2019). 30784830 10.1016/j.envpol.2019.01.120

[12] Rueda-López, I., Trejo-Téllez, L. I., Gómez-Merino, F. C., Peralta-Sánchez, M. G. & Ramírez-Olvera, S. M. Neodymium and zinc stimulate growth, biomass accumulation and nutrient uptake of lettuce plants in hydroponics. *Folia Hortic.* **36**(2), 283–297. 10.2478/fhort-2024-0017 (2024).

[13] Ozturk, M. et al. Role of Rare Earth Elements in Plants. *Plant Mol. Biol. Rep.* **41**(3), 345–368. 10.1007/s11105-023-01369-7 (2023).

[14] Zhang, C., Li, Q., Zhang, M., Zhang, N. & Li, M. Effects of rare earth elements on growth and metabolism of medicinal plants. *Acta Pharmaceutica Sinica B* **3**(1), 20–24. 10.1016/j.apsb.2012.12.005 (2013).

[15] Agra, M. F., Baracho, G. S., Nurit, K., Basílio, I. J. L. D. & Coelho, V. P. M. Medicinal and poisonous diversity of the flora of “Cariri Paraibano” Brazil. *J. of Ethnopharmacol.* **111**(2), 383–395. 10.1016/j.jep.2006.12.007 (2007). 17236731 10.1016/j.jep.2006.12.007

[16] Ding, N. et al. Physalin D regulates macrophage M1/M2 polarization via the STAT1/6 pathway. *J. Cell. Physiol.* **234**(6), 8788–8796. 10.1002/jcp.27537 (2019). 30317606 10.1002/jcp.27537

[17] Lin, Y.-H. et al. Physalin A attenuates inflammation through down-regulating c-Jun NH2 kinase phosphorylation/Activator Protein 1 activation and up-regulating the antioxidant activity. *Toxicol. Appl. Pharmacol.* **402**, 115115. 10.1016/j.taap.2020.115115 (2020). 32634518 10.1016/j.taap.2020.115115

[18] Soares, M. B. P., Bellintani, M. C., Ribeiro, I. M., Tomassini, T. C. B. & Ribeiro Dos Santos, R. Inhibition of macrophage activation and lipopolysaccaride-induced death by seco-steroids purified from *Physalis angulata* L. *Eur. J. Pharmacol.* **459**(1), 107–112. 10.1016/s0014-2999(02)02829-7 (2003). 12505539 10.1016/s0014-2999(02)02829-7

[19] Yang, Y. Anti-inflammatory effects of physalin E from *Physalis angulata* on lipopolysaccharide-stimulated RAW 264.7 cells through inhibition of NF-κB pathway. *Immunopharmacol. Immunotoxicol.* **39**(2), 74–79. 10.1080/08923973.2017.1282514 (2017). 28152630 10.1080/08923973.2017.1282514

[20] Ozaslan, C. et al. Germination Biology of Two Invasive Physalis Species and Implications for Their Management in Arid and Semi-arid Regions. *Sci. Rep.* **7**(1), 16960. 10.1038/s41598-017-17169-5 (2017). 29208989 10.1038/s41598-017-17169-5 PMC5717255

[21] Torres Tanan, T., Da Leite Silva, A., Da Silva Leite, R., Soares Arriero, S. & Neves do Nascimento, M. Physalis growth, development and yield at different sowing seasons in the brazilian northeastern semiarid. In: COLLOQ AGRARIAE **17**(1), 36–43. 10.5747/ca.2021.v17.n1.a418. (2021).

[22] Hayashi, T. Biologically active diterpenoids from scoparia dulcis l. (scrophulariaceae). In *Studies in Natural Products Chemistry* Vol. vol. 21 689–727 (Elsevier, 2000).

[23] Freire, S. M., Torres, L. M., Souccar, C. & Lapa, A. J. Sympathomimetic effects of Scoparia dulcis L. and catecholamines isolated from plant extracts. *The J. of Pharm. and Pharmacol.* **48**(6), 624–628. 10.1111/j.2042-7158.1996.tb05985.x (1996). 8832498 10.1111/j.2042-7158.1996.tb05985.x

[24] Ahmed, R., Ibrahim, H., Yakubu, M. & Dickson, P. Phytochemical and Anti-Inflammatory Studies on Methanol Leaf Extract of Scoparia Dulcis Linn. *Afr. J. Biomed. Res.*10.4314/ajbr.v25i2.21 (2023).

[25] Lima, G. M. et al. Assessment of antinociceptive and anti-inflammatory activities of Porophyllum ruderale (Jacq.) Cass., Asteraceae, aqueous extract. *Rev. Bras. Farmacogn.* **21**(3), 486–490. 10.1590/S0102-695X2011005000051 (2011).

[26] Tsai, J. Anti-inflammatory effects of *Scoparia dulcis* L. and betulinic acid. *Am. J. Chin. Med.* **39**(5), 943–956. 10.1142/S0192415X11009329 (2011). 21905284 10.1142/S0192415X11009329

[27] Nur-e-Alam, M. et al. Isolation and characterization of cytotoxic and anti-inflammatory constituents from Scoparia dulcis L. *J. Chem. Res.* **44**(7–8), 381–387. 10.1177/1747519819901100 (2020).

[28] Hayashi T. Scoparia dulcis L. (Sweet Broomweed): In Vitro Culture and the Production of Diterpenoids and Other Secondary Metabolites. In: Medicinal and Aromatic Plants IX. Springer, Berlin, Heidelberg, 370–383. (1996).

[29] Pawłowska, K. A. et al. The contribution of phenolics to the anti-inflammatory potential of the extract from Bolivian coriander (Porophyllum ruderale subsp. ruderale). *Food Chem.* **371**, 131116. 10.1016/j.foodchem.2021.131116 (2022). 34583181 10.1016/j.foodchem.2021.131116

[30] Marques, É. A., Oliveira, J. A., Coelho, A. D., Salimena, J. P. & Gavilanes, M. L. Porophyllum ruderale (Jacq.) Cass. uma revisão dos últimos 39 anos. *RSD* **9**(7), e944975215. 10.33448/rsd-v9i7.5215 (2020).

[31] Vargas-Madriz, Á. F. et al. Comparison of phenolic compounds and evaluation of antioxidant properties of *Porophyllum ruderale* (Jacq.) Cass (Asteraceae) from different geographical areas of Queretaro (Mexico). *Plants* **12**(20), 3569. 10.3390/plants12203569 (2023). 37896032 10.3390/plants12203569 PMC10609970

[32] Plants of the World Online. Porophyllum ruderale (Jacq.) Cass. | Plants of the World Online | Kew Science. https://powo.science.kew.org/taxon/urn:lsid:ipni.org:names:208446-2/general-information (accessed 5 December 2025). (2025)

[33] Chanput, W., Mes, J. J. & Wichers, H. J. THP-1 cell line: an in vitro cell model for immune modulation approach. *Int. Immunopharmacol.* **23**(1), 37–45. 10.1016/j.intimp.2014.08.002 (2014). 25130606 10.1016/j.intimp.2014.08.002

[34] Cai, Y. et al. Physalin H ameliorates LPS-induced acute lung injury via KEAP1/NRF2 axis. *Int. Immunopharmacol.* **131**, 111789. 10.1016/j.intimp.2024.111789 (2024). 38484668 10.1016/j.intimp.2024.111789

[35] Liu, Q. et al. Bioactive diterpenoids and flavonoids from the aerial parts of Scoparia dulcis. *J. Nat. Prod.* **77**(7), 1594–1600. 10.1021/np500150f (2014). 24955889 10.1021/np500150f

[36] ISO, 1994. ISO 11265: Soil quality — Determination of the specific electrical conductivity. https://www.iso.org/standard/19243.html (accessed 28 April 2025).

[37] Analytik Jena. Determination of TOC in Agricultural Soil, Dried Manure and Sediments. https://www.analytik-jena.com/import/assets/12562450_AppNote_SP_0019_en_multiNC_TOC_agricultural_soil_sediments.pdf (accessed 28 April 2025). (2025).

[38] Durner, W., Iden, S. C. & von Unold, G. The integral suspension pressure method (ISP ) for precise particle-size analysis by gravitational sedimentation. *Water Resour. Res.* **53**(1), 33–48. 10.1002/2016WR019830 (2017).

[39] Malathi, P. & Stalin, P. Evaluation of AB - DTPA Extractant for Multinutrients Extraction in Soils. *Int. J. Curr. Microbiol. App. Sci.* **7**(03), 1192–1205. 10.20546/ijcmas.2018.703.141 (2018).

[40] Dührkop, K. et al. SIRIUS 4: a rapid tool for turning tandem mass spectra into metabolite structure information. *Nat Methods* **16**(4), 299–302. 10.1038/s41592-019-0344-8 (2019). 30886413 10.1038/s41592-019-0344-8

[41] Ludwig, M. et al. Database-independent molecular formula annotation using Gibbs sampling through ZODIAC. *Nat. Mach. Intell.* **2**(10), 629–641. 10.1038/s42256-020-00234-6 (2020).

[42] Dührkop, K., Shen, H., Meusel, M., Rousu, J. & Böcker, S. Searching molecular structure databases with tandem mass spectra using CSI:FingerID. *Proc. Natl. Acad. Sci. U.S.A.* **112**(41), 12580–12585. 10.1073/pnas.1509788112 (2015). 26392543 10.1073/pnas.1509788112 PMC4611636

[43] Shen, H., Dührkop, K., Böcker, S. & Rousu, J. Metabolite identification through multiple kernel learning on fragmentation trees. *Bioinformatics* **30**(12), i157–i164. 10.1093/bioinformatics/btu275 (2014). 24931979 10.1093/bioinformatics/btu275 PMC4058957

[44] Dührkop, K. et al. Systematic classification of unknown metabolites using high-resolution fragmentation mass spectra. *Nat Biotechnol* **39**(4), 462–471. 10.1038/s41587-020-0740-8 (2021). 33230292 10.1038/s41587-020-0740-8

[45] Kim, H. W. et al. NPClassifier: A Deep Neural Network-Based Structural Classification Tool for Natural Products. *J. Nat. Prod.* **84**(11), 2795–2807. 10.1021/acs.jnatprod.1c00399 (2021). 34662515 10.1021/acs.jnatprod.1c00399 PMC8631337

[46] Kome, G. K., Enang, R. K., Tabi, F. O. & Yerima, B. P. K. Influence of Clay Minerals on Some Soil Fertility Attributes: A Review. *OJSS* **09**(09), 155–188. 10.4236/ojss.2019.99010 (2019).

[47] Singh, M., Sarkar, B., Biswas, B., Bolan, N. S. & Churchman, G. J. Relationship between soil clay mineralogy and carbon protection capacity as influenced by temperature and moisture. *Soil Biol. Biochem.* **109**, 95–106. 10.1016/j.soilbio.2017.02.003 (2017).

[48] Cheng, Y. et al. Spatial distribution characteristics and pollution evaluation of soil iron in the Middle Hanjiang River. *Int. J. Environ. Res. Public Health* **16**(21), 4075. 10.3390/ijerph16214075 (2019). 31652749 10.3390/ijerph16214075 PMC6862237

[49] Li, H. et al. Soil texture controls added organic matter mineralization by regulating soil moisture—evidence from a field experiment in a maritime climate. *Geoderma* **410**, 115690. 10.1016/j.geoderma.2021.115690 (2022).

[50] Poeplau, C., Dechow, R., Begill, N. & Don, A. Towards an ecosystem capacity to stabilise organic carbon in soils. *Glob. Change Biol.* **30**(8), e17453. 10.1111/gcb.17453 (2024). 10.1111/gcb.17453 39099457

[51] Pikuła, D. & Stępień, W. Effect of the Degree of Soil Contamination with Heavy Metals on Their Mobility in the Soil Profile in a Microplot Experiment. *Agronomy* **11**(5), 878. 10.3390/agronomy11050878 (2021).

[52] Król, A., Mizerna, K. & Bożym, M. An assessment of pH-dependent release and mobility of heavy metals from metallurgical slag. *J. Hazard. Mater.* **384**, 121502. 10.1016/j.jhazmat.2019.121502 (2020). 31732354 10.1016/j.jhazmat.2019.121502

[53] Sintorini, M. M., Widyatmoko, H., Sinaga, E. & Aliyah, N. Effect of pH on metal mobility in the soil. IOP Conf. Ser. Earth Environ. Sci. 737 (1), 12071. 10.1088/1755-1315/737/1/012071. (2021).

[54] Harrison, A. F. *Soil organic phosphorus: A review of world literature* 257 (CAB International, 1987).

[55] Mengel, K., Kirkby, E. A., Kosegarten, H. & Appel, T. Magnesium. In *Principles of Plant Nutrition* 5th edn (eds Mengel, K. et al.) 541–552 (Springer, 2001).

[56] Smith, D. B., Solano, F., Woodruff, L. G., Cannon, W. F. & Ellefsen, K. J. Scientific Investigations Report. (2019).

[57] Rakkar, M. K., Franzen, D. W. & Chatterjee, A. Evaluation of Soil Potassium Test to Improve Fertilizer Recommendations for Corn. *OJSS* **05**(05), 110–122. 10.4236/ojss.2015.55011 (2015).

[58] Breker, J. S. et al. Potassium Requirements for Corn in North Dakota: Influence of Clay Mineralogy. *Soil Sci. Soc. of Amer. J.* **83**(2), 429–436. 10.2136/sssaj2018.10.0376 (2019).

[59] Yesmin, R. et al. Evaluation of critical limit of sulphur in soils for wheat (*Triticum aestivum* L.) and mustard (*Brassica napus* L.). *Sustainability* **13**(15), 8325. 10.3390/su13158325 (2021).

[60] Power, J. F. & Prasad, R. *Soil Fertility Management for Sustainable Agriculture* (CRC Press, 1997).

[61] Singh, S., Nath, A., Shukla S. & Tripathi, K. M. Potassium Management Strategy to Increase Potassium use Efficiency (KUE). (2024)

[62] Stanton, C., Sanders, D., Krämer, U. & Podar, D. Zinc in plants: Integrating homeostasis and biofortification. *Mol. Plant* **15**(1), 65–85. 10.1016/j.molp.2021.12.008 (2022). 34952215 10.1016/j.molp.2021.12.008

[63] Meng, Y. et al. Toxicity effects of zinc supply on growth revealed by physiological and transcriptomic evidences in sweet potato (Ipomoea batatas (L.) Lam). *Sci. Rep.* **13**(1), 19203. 10.1038/s41598-023-46504-2 (2023). 37932351 10.1038/s41598-023-46504-2 PMC10628244

[64] El-Ramady, H. R. et al. Soil Quality and Plant Nutrition. *Sustain. Agric. Rev.* **14**(14), 345–447. 10.1007/978-3-319-06016-3_11 (2014).

[65] Fukalova Fukalova, T., García-Martínez, M. D. & Raigón, M. D. Nutritional Composition, Bioactive Compounds, and Volatiles Profile Characterization of Two Edible Undervalued Plants: Portulaca oleracea L. and Porophyllum ruderale (Jacq.) Cass. *Plants* **11**(3), 377. 10.3390/plants11030377 (2022). 35161358 10.3390/plants11030377 PMC8839399

[66] Matteo, M., Zoffoli, J. P., van der Heijden, G. & Ayala, M. Calcium absorption by fruit and leaves of sweet cherry trees (Prunus avium L.) by isotope labeling. *Sci. Hortic.* **329**, 113026 (2024).

[67] Penot, M. & Floc’h, J. Y. Étude comparée de l’Absorption et de la rédistribution du (45)Ca chez divers groupes de végétaux. *Planta* **129**(1), 7–14. 10.1007/BF00390905 (1976). 24430807 10.1007/BF00390905

[68] Lapaz, A. Iron toxicity: effects on the plants and detoxification strategies. *Acta Bot. Bras.*10.1590/0102-33062021abb0131 (2022).

[69] Batty, L. C. & Younger, P. L. Effects of external iron concentration upon seedling growth and uptake of Fe and phosphate by the common reed, Phragmites australis (Cav.) Trin ex Steudel. *Ann. of Bot.* **92**(6), 801–806 (2003). 14565939 10.1093/aob/mcg205 PMC4243622

[70] Harish, V., Aslam, S., Chouhan, S., Pratap, Y. & Lalotra, S. Iron toxicity in plants: A Review. *IJECC* **13**(8), 1894–1900. 10.9734/IJECC/2023/v13i82145 (2023).

[71] Zahra, N., Hafeez, M. B., Shaukat, K., Wahid, A. & Hasanuzzaman, M. Fe toxicity in plants: Impacts and remediation. *Physiol. Plant.* **173**(1), 201–222. 10.1111/ppl.13361 (2021). 33547807 10.1111/ppl.13361

[72] Hassan, M. U. et al. Nickel toxicity in plants: reasons, toxic effects, tolerance mechanisms, and remediation possibilities-a review. *Environ. Sci. Pollut. Res.* **26**(13), 12673–12688. 10.1007/s11356-019-04892-x (2019). 10.1007/s11356-019-04892-x 30924044

[73] Mao, X. et al. Microbial assisted alleviation of nickel toxicity in plants: A review. *Ecotoxicol. Environ. Saf.* **289**, 117669. 10.1016/j.ecoenv.2025.117669 (2025). 39788037 10.1016/j.ecoenv.2025.117669

[74] Cruz, F., Ferreira, R. Conceição, S., Lima, E., Neto, C.F., Rodrigues Galvão, J., Lopes, S. & Viégas, I. Copper Toxicity in Plants: Nutritional, Physiological, and Biochemical Aspects. In: (2022).

[75] Shabbir, Z. et al. Copper uptake, essentiality, toxicity, detoxification and risk assessment in soil-plant environment. *Chemosphere* **259**, 127436. 10.1016/j.chemosphere.2020.127436 (2020). 32599387 10.1016/j.chemosphere.2020.127436

[76] Xie, K., Cakmak, I., Wang, S., Zhang, F. & Guo, S. Synergistic and antagonistic interactions between potassium and magnesium in higher plants. *The Crop J.* **9**(2), 249–256. 10.1016/j.cj.2020.10.005 (2021).

[77] Arévalo-Hernández, C. O., Nascimento Junior, A. L., Queiroz, A. P., Gross, E. & Da Souza, L. S. Exploratory analysis of trace elements in soils and plants affected by a gossan in the Semiarid. *Rev. Bras. Eng. Agríc. Ambient.* **25**(2), 139–145. 10.1590/1807-1929/agriambi.v25n2p139-145 (2021).

[78] Ahsan, M., Islam, S. K. N., Gray, A. I. & Stimson, W. H. Cytotoxic diterpenes from Scoparia dulcis. *J. Nat. Prod.* **66**(7), 958–961. 10.1021/np020356j (2003). 12880314 10.1021/np020356j

[79] Fuentes, R. G. et al. Scopadulciol, Isolated from Scoparia dulcis, Induces β-Catenin Degradation and Overcomes Tumor Necrosis Factor-Related Apoptosis Ligand Resistance in AGS Human Gastric Adenocarcinoma Cells. *J. Nat. Prod.* **78**(4), 864–872. 10.1021/np500933v (2015). 25793965 10.1021/np500933v

[80] Li, Q., Gao, Y. & Yang, an. Sulfur Homeostasis in Plants. *Int. J. Mol. Sci.* **21**(23), 8926. 10.3390/ijms21238926 (2020). 33255536 10.3390/ijms21238926 PMC7727837

[81] Miękus, N. et al. Health Benefits of Plant-Derived Sulfur Compounds, Glucosinolates, and Organosulfur Compounds. *Molecules* **25**(17), 3804. 10.3390/molecules25173804 (2020). 32825600 10.3390/molecules25173804 PMC7503525

[82] Van, H.-T., van Hoang, H., Nga, L. T. Q. & van Nguyen, Q. Effects of Zn pollution on soil: Pollution sources, impacts and solutions. *Results in Surf. and Interfaces* **17**, 100360. 10.1016/j.rsurfi.2024.100360 (2024).

[83] Feng, X., Zhu, G. & LI, Y. Toxicological effects of rare earth yttrium on wheat seedlings (Triticum aestivum). *J. of Rare Earths* **31**(12), 1214–1220. 10.1016/s1002-0721(12)60429-3 (2013).

[84] Agathokleous, E. et al. Mechanisms of cerium-induced stress in plants: A meta-analysis. *The Sci. of the Total Environ.* **852**, 158352. 10.1016/j.scitotenv.2022.158352 (2022). 10.1016/j.scitotenv.2022.158352 36063950

[85] Rezaee, A., Hale, B., Santos, R. M. & Chiang, Y. W. Accumulation and toxicity of lanthanum and neodymium in horticultural plants (Brassica chinensis L. and Helianthus annuus L.). *Can. J. Chem. Eng.* **96**(10), 2263–2272 (2018).

[86] Forsyth, K. et al. Bioconcentration and translocation of rare earth elements in plants collected from three legacy mine sites in Portugal. *Front. Environ. Sci.* **11**, 1191909. 10.3389/fenvs.2023.1191909 (2023).

[87] Kastori, R. R., Maksimović, I. V. & Putnik-Delić, M. I. Rare earth elements in environment and effects on plants: A review scientific paper. *Zbornik Matice srpske za prirodne nauke* **144**, 51–72 (2023).

[88] Guo, Y. et al. Rare earth elements (rees) adsorption and detoxification mechanisms in cell wall polysaccharides of Phytolacca americana L. *Plants* **12**(10), 1981. 10.3390/plants12101981 (2023). 37653898 10.3390/plants12101981 PMC10223583

[89] Shan, X. et al. Accumulation and uptake of light rare earth elements in a hyperaccumulator Dicropteris Dichotoma. *Plant Sci.* **165**(6), 1343–1353. 10.1016/s0168-9452(03)00361-3 (2003).

[90] Abu Darwish, M. S. Essential oil variation and trace metals content in garden sage (salvia officinalis L.) grown at different environmental conditions. *J. of Agric. Sci.* **6**(3), 209 (2014).

[91] Zheljazkov, V. & Jekov, D. Heavy metal content in some essential oils and plant extracts. *Acta Hort.* **426**, 427–434. 10.17660/actahortic.1996.426.47 (1996).

[92] Doulgeridou, A., Amlund, H., Sloth, J. J. & Hansen, M. Review of potentially toxic rare earth elements, thallium and tellurium in plant-based foods. *EFSA J.*10.2903/j.efsa.2020.e181101 (2020). 33294040 10.2903/j.efsa.2020.e181101 PMC7691615

[93] Franchi, L., Eigenbrod, T., Muñoz-Planillo, R. & Nuñez, G. The inflammasome: a caspase-1-activation platform that regulates immune responses and disease pathogenesis. *Nat Immunol* **10**(3), 241–247. 10.1038/ni.1703 (2009). 19221555 10.1038/ni.1703 PMC2820724

[94] Martinon, F., Burns, K. & Tschopp, J. The inflammasome: a molecular platform triggering activation of inflammatory caspases and processing of proIL-beta. *Mol. Cell* **10**(2), 417–426. 10.1016/s1097-2765(02)00599-3 (2002). 12191486 10.1016/s1097-2765(02)00599-3

[95] Li, Y.-P., Wu, D.-X., Ye, T. & Zhang, H. Cytotoxic diterpenoids from the aerial parts of Scoparia dulcis. *Phytochem. Lett.* **49**, 21–26. 10.1016/j.phytol.2022.02.014 (2022).

[96] Wong, J., Tran, L. T., Magun, E. A., Magun, B. E. & Wood, L. J. Production of IL-1β by bone marrow-derived macrophages in response to chemotherapeutic drugs: synergistic effects of doxorubicin and vincristine. *Cancer Biol. Ther.* **15**(10), 1395–1403. 10.4161/cbt.29922 (2014). 25046000 10.4161/cbt.29922 PMC4130732

[97] Lima, M. V. V. & Freire, A. Therapeutic Use of Scoparia dulcis Reduces the Progression of Experimental Osteoarthritis. *Molecules*10.3390/molecules24193474 (2019). 31557835 10.3390/molecules24193474 PMC6803828

