# Correction: German soils affect biomass production, elemental profiles, and anti-inflammatory activity of three medicinal plants used in Brazilian traditional medicine: *Scoparia dulcis* L., *Physalis angulata *L., and *Porophyllum ruderale* (Jacq.) Cass.

**DOI:** 10.1038/s41598-026-64028-3

**Published:** 2026-07-27

**Authors:** Enrique B. Hernández, Philipp Gabor, Franziska Schanbacher, Vanessa Gisele Pasqualotto Severino, Wilson Mozena Leandro, Sabry M. Shaheen, Matthias Melzig, Alexander Weng, Jörg Rinklebe

**Affiliations:** 1 School of Architecture and Civil Engineering, Institute of Foundation Engineering, Water- and Waste-Management, Laboratory of Soil- and Groundwater-Management, University of Wuppertal, Pauluskirchstraße 7, 42285 Wuppertal, Germany; 2 Institute of Pharmacy, Freie Universität Berlin, Königin-Luise-Str. 2+4, 14195 Berlin, Germany; 3 Federal University of Goiás, University Campus, Esperança Avenue, Goiânia, Brazil

Correction to: *Scientific Reports* 10.1038/s41598-026-56323-w, published online 18 June 2026

The original version of this Article contained an error in the Reference List. The authors omitted the below Reference, which is now listed as Reference 10.

Vilela, L., Ramos, S., Carneiro, M. A., Faquin, V., Guilherme, L., Siqueira, O. 2018. Cerium (Ce) and Lanthanum (La) promoted plant growth and mycorrhizal colonization of maize in tropical soil. Australian Journal of Crop Science. 12. 704-710. 10.21475/ajcs.18.12.05.PNE754.

As a result, in the Introduction,

“In low concentrations (~ 40 mg dm−3), these two elements have also been shown to support mycorrhizal relationships in the rhizosphere of some species, as a result showing significant improvement in plant growth^9^.”

now reads:

“In low concentrations (~ 40 mg dm−3), these two elements have also been shown to support mycorrhizal relationships in the rhizosphere of some species, as a result showing significant improvement in plant growth^9,10^.”

As a result of the changes, the References have been renumbered.

The original Article has been corrected.

