# Supplementary material for: German soils affect biomass production, elemental profiles, and anti-inflammatory activity of three medicinal plants used in Brazilian traditional medicine: Scoparia dulcis L., Physalis angulata L., and Porophyllum ruderale (Jacq.) Cass
Source: Sci Rep. 2026 Jun 18;16:19023. doi: 10.1038/s41598-026-56323-w (PMC13279939; doi:10.1038/s41598-026-56323-w)
Supplement: Supplementary file 1 — Supplementary Information. [file 41598_2026_56323_MOESM1_ESM.docx]

**German soils affect biomass production, elemental profiles, and anti-inflammatory activity of the Brazilian plants *Scoparia dulcis* L., *Physalis angulata* L., and *Porophyllum ruderale* (Jacq.) Cass..**

**Enrique B. Hernández ^a †^, Philipp Gabor ^b †^, Franziska Schanbacher ^b^,** **Vanessa Gisele Pasqualotto Severino^c^,** **Wilson Mozena Leandro ^c^,** **Sabry M. Shaheen ^a^, Matthias Melzig ^b^, Alexander Weng ^b *^, Jörg Rinklebe ^a *^**

^a^ University of Wuppertal, School of Architecture and Civil Engineering, Institute of Foundation Engineering, Water- and Waste-Management, Laboratory of Soil- and Groundwater-Management, Pauluskirchstraße 7, 42285 Wuppertal, Germany.

^b^ Freie Universität Berlin, Institute of Pharmacy, Königin-Luise-Str. 2+4, D-14195 Berlin, Germany.

^c^  Federal University of Goiás, Esperança Avenue, University Campus, Goiânia, Brazil

**^†^** These authors share first authorship and contributed equally to this work

* Corresponding authors:

**Jörg Rinklebe** Email address: [rinklebe@uni-wuppertal.de](mailto:rinklebe@uni-wuppertal.de)

**Alexander Weng** Email address: [alexander.weng@fu-berlin.de](mailto:alexander.weng@fu-berlin.de)

1. The Bioconcentration Factor (BCF) and the Translocation Factor (TF) were calculated by using the following equations:

$$BCF=\frac{Element concentration in roots (mg kg-1)}{Element concentration in soil (mg kg-1)}$$

$$TF=\frac{Element concentration in stems or in leaves (mg kg-1)}{Element concentration in roots (mg kg-1)}$$

| **Tab. S1.** Bioconcentration Factor and Translocation Factor calculated in *Porophyllum ruderale* (Jacq) Cass., *Scoparia dulcis* L. and *Physalis angulata* L. grown in clayey silt soil. | | | | | | | | | | | | | | | |
| --- | --- | --- | --- | --- | --- | --- | --- | --- | --- | --- | --- | --- | --- | --- | --- |
|  | | *P. ruderale* | | | | | *S. dulcis* | | | | | *P. angulata* | | | |
| Elements | | Bioacumulation factor | | Translocation Factor in Leaves | | Translocation Factor in Stems | Bioacumulation factor | | Translocation Factor in Leaves | | Translocation Factor in Stems | Bioacumulation factor | | Translocation Factor in Leaves | Translocation Factor in Stems |
| Cu | | 0.60 | | 0.30 | | 0.42 | 0.41 | | 1.26 | | 0.99 | 1.24 | | 0.37 | 0.59 |
| Fe | | 0.04 | | 0.04 | | 0.03 | 0.04 | | 0.08 | | 0.04 | 0.05 | | 0.06 | 0.04 |
| Mn | | 0.20 | | 0.15 | | 0.03 | 0.04 | | 0.99 | | 0.36 | 0.04 | | 0.43 | 0.07 |
| Ni | | 0.10 | | 0.44 | | 0.63 | 0.09 | | 0.58 | | 0.71 | 0.10 | | 0.64 | 0.48 |
| Zn | | 0.34 | | 0.29 | | 0.29 | 0.60 | | 0.45 | | 0.47 | 0.34 | | 0.31 | 0.93 |
| S | | 6.19 | | 0.34 | | 0.28 | 2.26 | | 0.56 | | 0.98 | 2.77 | | 4.48 | 3.38 |
| P | | 2.67 | | 0.73 | | 0.37 | 3.09 | | 1.28 | | 0.51 | 2.82 | | 1.63 | 1.50 |
| Mg | | 0.40 | | 0.51 | | 0.23 | 0.29 | | 1.09 | | 0.55 | 0.25 | | 2.93 | 1.08 |
| K | | 0.33 | | 0.49 | | 0.29 | 0.26 | | 1.02 | | 0.60 | 0.23 | | 2.54 | 1.29 |
| Ca | | 0.43 | | 6.53 | | 0.68 | 0.23 | | 3.46 | | 3.48 | 0.34 | | 4.84 | 2.04 |
| Y | | 0.05 | | 0.05 | | 0.02 | 0.03 | | 0.10 | | 0.04 | 0.04 | | 0.07 | 0.05 |
| La | | 0.03 | | 0.06 | | 0.02 | 0.02 | | 0.09 | | 0.05 | 0.03 | | 0.07 | 0.04 |
| Ce | | 0.03 | | 0.04 | | 0.04 | 0.02 | | 0.09 | | 0.07 | 0.03 | | 0.07 | 0.04 |
| Nd | | 0.03 | | 0.05 | | 0.02 | 0.02 | | 0.09 | | 0.04 | 0.03 | | 0.06 | 0.03 |
|  | |  | |  | |  |  | |  | |  |  | |  |  |
|  | |  | |  | |  |  | |  | |  |  | |  |  |
| **Tab. S2.** Bioconcentration Factor and Translocation Factor calculated in *Porophyllum ruderale* (Jacq) Cass., *Scoparia dulcis* L. and *Physalis angulata* L. grown in sandy loam soil. | | | | | | | | | | | | | | | |
|  | *P. ruderale* | | | | | | *S. dulcis* | | | | | *P. angulata* | | | |
| Elements | Bioacumulation factor | | Translocation Factor in Leaves | | Translocation Factor in Stems | | Bioacumulation factor | Translocation Factor in Leaves | | Translocation Factor in Stems | | Bioacumulation factor | Translocation Factor in Leaves | | Translocation Factor in Stems |
| Cu | 0.05 | | 0.30 | | 0.11 | | 0.02 | 0.85 | | 0.89 | | 0.04 | 0.30 | | 0.30 |
| Fe | 0.42 | | 0.07 | | 0.03 | | 0.36 | 0.08 | | 0.05 | | 0.45 | 0.07 | | 0.05 |
| Mn | 0.01 | | 2.61 | | 0.28 | | 0.02 | 1.44 | | 0.71 | | 0.02 | 0.66 | | 0.12 |
| Ni | 1.21 × 10^-3^ | | 0.66 | | 0.62 | | 0.00 | 0.79 | | 0.85 | | 9.2 × 10^-4^ | 0.66 | | 0.97 |
| Zn | 4.38 | | 22.10 | | 8.53 | | 3.90 | 9.03 | | 11.30 | | 3.14 | 68.67 | | 108.56 |
| S | 185.00 | | 3.8 × 10^-3^ | | 3.2 × 10^-3^ | | 37.85 | 0.07 | | 0.06 | | 63.83 | 0.01 | | 0.05 |
| P | 0.11 | | 0.74 | | 0.54 | | 0.12 | 1.55 | | 0.80 | | 0.29 | 0.57 | | 1.21 |
| Mg | 0.87 | | 1.10 | | 0.52 | | 1.57 | 1.10 | | 0.66 | | 1.23 | 2.03 | | 1.19 |
| K | 1.82 | | 1.19 | | 0.61 | | 3.30 | 1.11 | | 0.73 | | 2.18 | 2.15 | | 1.64 |
| Ca | 9.38 | | 10.34 | | 1.30 | | 10.59 | 2.66 | | 2.94 | | 12.81 | 3.38 | | 1.82 |
| Y | 0.05 | | 0.11 | | 0.03 | | 0.03 | 0.11 | | 0.06 | | 0.04 | 0.13 | | 0.07 |
| La | 0.06 | | 0.07 | | 0.02 | | 0.06 | 0.04 | | 0.02 | | 0.07 | 0.05 | | 0.02 |
| Ce | 0.04 | | 0.12 | | 0.03 | | 0.03 | 0.07 | | 0.06 | | 0.04 | 0.06 | | 0.04 |
| Nd | 0.06 | | 0.08 | | 0.02 | | 0.05 | 0.05 | | 0.03 | | 0.07 | 0.05 | | 0.02 |
|  |  | |  | |  | |  |  | |  | |  |  | |  |
|  |  | |  | |  | |  |  | |  | |  |  | |  |

**
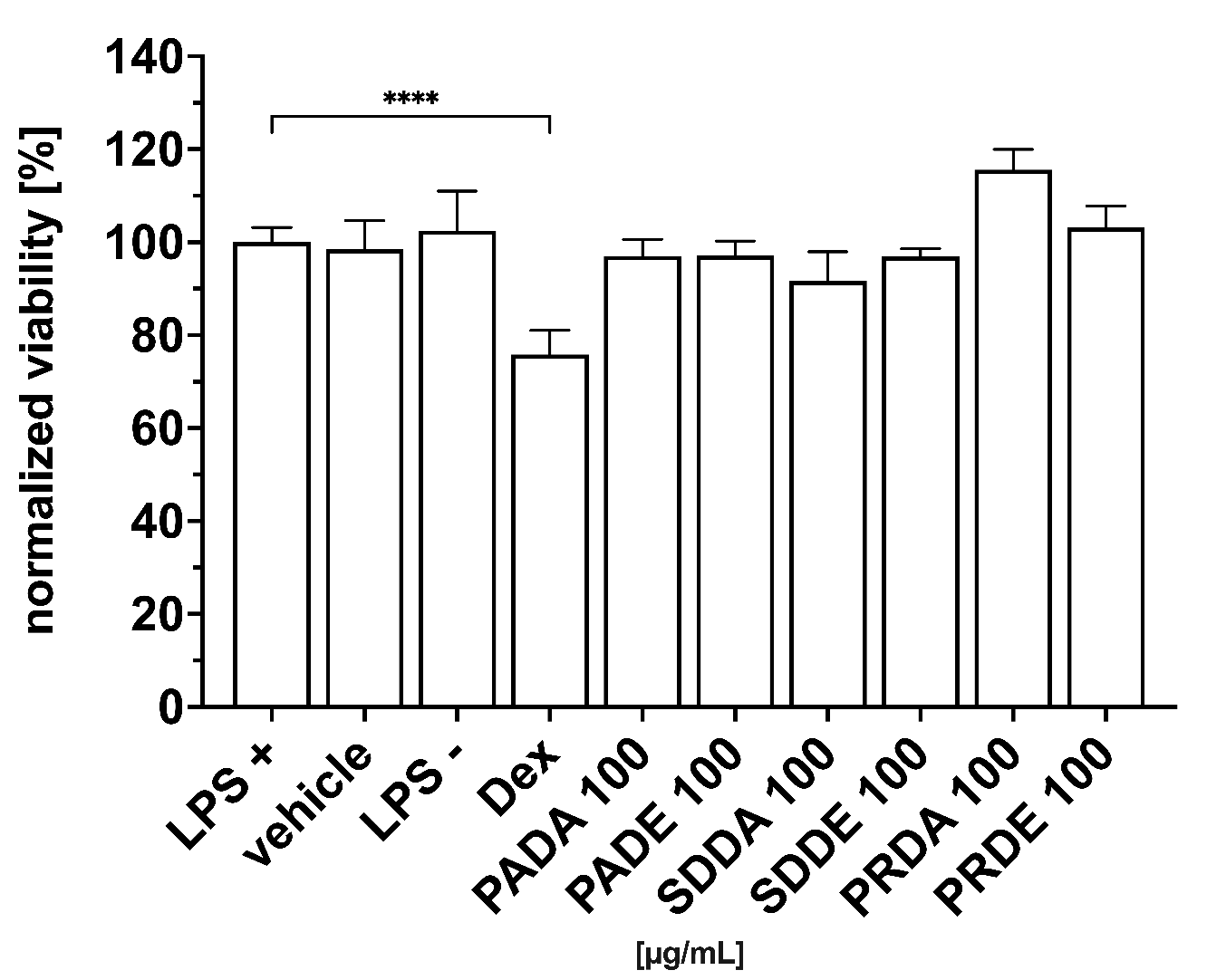
**

**Fig S1:** Viability of lipopolysaccharide (LPS) stimulated PMA-differentiated THP-1 cells 24h after treatment using WST-8 assay. Data was normalized to the LPS + control and represents Mean ± SD of min. 3 replicates. Vehicle control = 0.1 % DMSO, Dex = 0,5 µM Dexamethasone, extract concentrations are in µg/mL. ****p<0.001 compared to the LPS+ control as determined by Kruskal-Wallis Test with post-hoc Dunn’s test. Data points without labelling did not significantly differ from LPS+


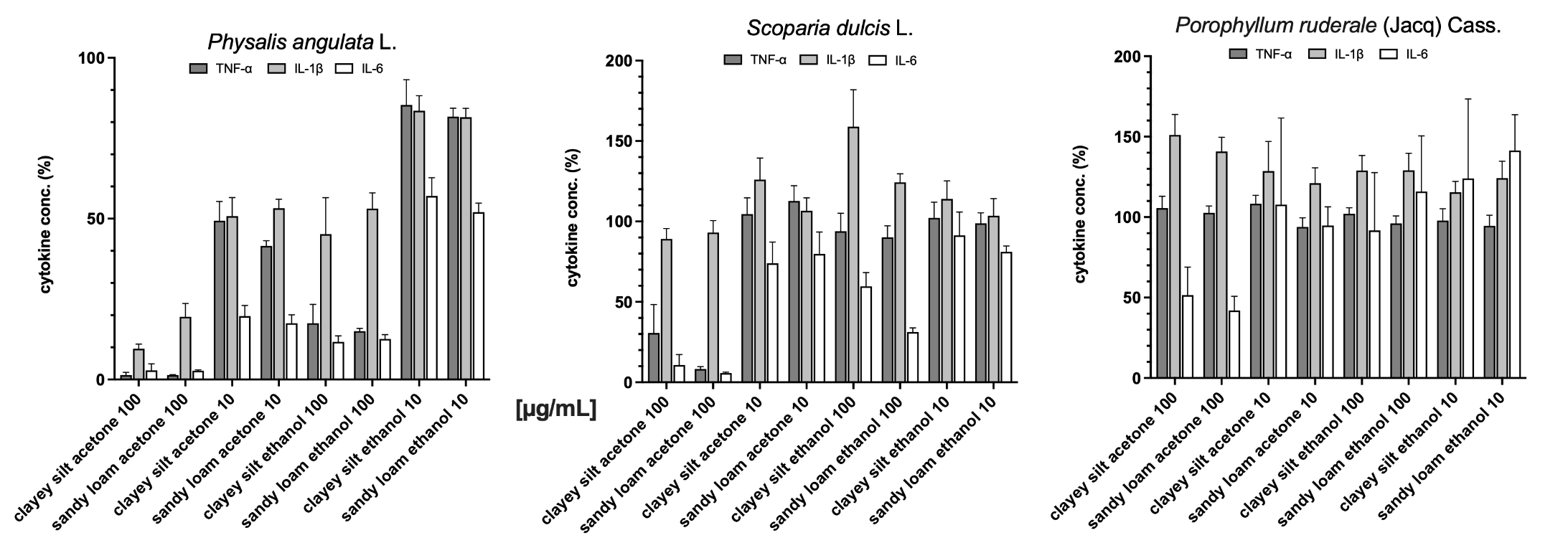


**Fig S2:** Impact of different soil types on cytokine production induced by different types of plant extracts. Data are normalized to the LPS+ control and represent mean ± SD from at least three replicates. Extract concentrations are given in µg/mL.
